# Structure and pH-Induced Swelling of Polymer Films Prepared from Sequentially Grafted Polyelectrolytes

**DOI:** 10.1021/acs.langmuir.1c02784

**Published:** 2022-01-26

**Authors:** Béla Nagy, Mario Campana, Yury N. Khaydukov, Thomas Ederth

**Affiliations:** † Division of Biophysics and Bioengineering, Department of Physics, Chemistry and Biology, Linköping University, SE-581 83 Linköping, Sweden; ‡ ISIS Facility, Rutherford Appleton Laboratory, STFC, Chilton, Didcot, Oxon OX11 0QX, U.K.; § Max-Planck-Institut für Festkörperforschung, Heisenbergstraße 1, D-70569 Stuttgart, Germany; ∥ Max Planck Society Outstation at the Heinz Maier-Leibnitz Zentrum (MLZ), D-85748 Garching, Germany

## Abstract

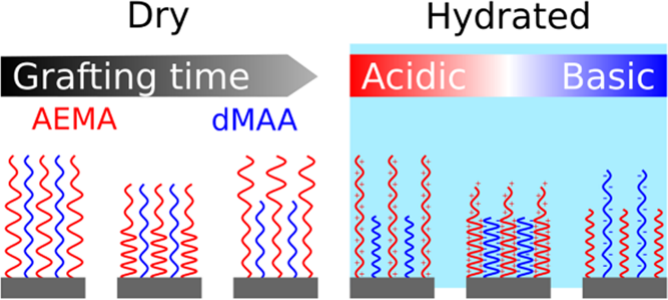

We have prepared a series of ampholytic polymer films, using a self-initiated photografting and photopolymerization (SI-PGP) method to sequentially polymerize first anionic (deuterated methacrylic acid (dMAA)) and thereafter cationic (2-aminoethyl methacrylate (AEMA)) monomers to investigate the SI-PGP grafting process. Dry films were investigated by ellipsometry, X-ray, and neutron reflectometry, and their swelling was followed over a pH range from 4.5 to 10.5 with spectroscopic ellipsometry. The deuterated monomer allows us to separate the distributions of the two components by neutron reflectometry. Growth of both polymers proceeds via grafting of solution-polymerized fragments to the surface, and also the second layer is primarily grafted to the substrate and not as a continuation of the existing chains. The polymer films are stratified, with one layer of near 1:1 composition and the other layer enriched in one component and located either above or below the former layer. The ellipsometry results show swelling transitions at low and high pH but with no systematic variation in the pH values where these transitions occur. The results suggest that grafting density in SI-PGP-prepared homopolymers could be increased via repeated polymerization steps, but that this process does not necessarily increase the average chain length.

## Introduction

The unwanted accumulation of biological material on surfaces is a concern in many fields of science and engineering, including marine environments,^1^ medical applications,^2^ and biosensing.^3^ Biofouling impairs the function and reduces the lifetime of installations and devices, causing both environmental damage and increased maintenance costs. Environmentally benign and biocompatible antifouling strategies frequently rely on physicochemical methods of fouling prevention, and various strongly hydrophilic coatings are widely used to this end. These prevent attachment by binding water molecules strongly to the surface, providing steric hindrance and increasing the enthalpic cost of attachment and in certain cases also rely on entropic effects acting upon the expulsion of water from hydrated polymer films to hinder macromolecular adsorption. This approach has a long history, with work on poly(2-hydroxyethyl methacrylate) (pHEMA) dating back over 60 years.^4^ Later on, interest in the field has expanded to cover a wide range of polymers and hydrogel materials,^5−7^ and considerable efforts have been spent in understanding and using poly(ethylene glycol) (PEG) for antifouling purposes due to its excellent fouling resistance.^8−10^ For PEG, the efficacy of strong hydration as a major reason for its antifouling properties has been qualitatively and quantitatively demonstrated^11^ and also explained, both at the molecular level^12,13^ and collectively for polymer brushes.^14^ Growing concerns about immunogenicity^15^ and stability^16^ of PEG are gradually shifting interest toward other polymers. In recent years, zwitterionic polymers have emerged as interesting candidates for antifouling applications.^17,18^ Being polyelectrolytes containing both anionic and cationic residues, zwitterionic polymers bind water efficiently because of the abundance of charged groups but do not participate in long-range Coulomb interactions due to their overall zero net charge. Most zwitterionic polymers are prepared from a very limited range of zwitterionic residues,^19^ but small differences in the molecular structure of the polymers can significantly influence their properties;^20−22^ hence, efforts are made to explore and understand the behavior and properties of different zwitterionic monomer structure variants.^19,23^

A greater variation in the type and ratio of charged groups may be achieved using pseudo-zwitterionic polymers. These are (often random) copolymers of anionic and cationic monomers. By using weak electrolytes as monomers, the net charge of the layers can be tuned by the solvent pH, opening possibilities for responsive surfaces and additional engineering of interfacial properties. In a series of papers, we have demonstrated and explored how thin polymer films prepared by sequential polymerization of anionic and cationic monomers can be used as powerful tools to investigate and exploit the pH dependence of such composite surfaces, to tune the net charge, and also the resistance to protein fouling.^24−26^ In these films, the second layer was grafted as a thickness gradient on top of a bottom layer of homogeneous thickness, using self-initiated photografting and photopolymerization (SI-PGP),^27^ which is a simple and robust method for creating polymer thin films from methacrylate monomers^28^ and which has been useful for the preparation of antifouling coatings.^10,29,30^ Fouling studies conducted on such sequentially grafted bilayer gradients have shown that resistance to nonspecific protein adsorption is optimal when the layer is charge-compensated, which is dependent on the pH and the location along the gradient.^24,25^ The charge equilibrium also coincides with a collapsed state of the polymer, leading to the unexpected observation that the most collapsed film is the most fouling-resistant, as opposed to nominally neutral polymers that are most fouling-resistant in the swollen state.^31^ While the utility of coatings prepared in this manner is demonstrated in the literature,^10,29,30,32^ further insights into the grafting and film formation are required to fully exploit this method. Correlating the antifouling properties to the swelling and the composition of the film at a given pH would allow for the development of coatings with switchable antifouling or pH-controllable cleaning functions. In a recent publication, we investigated the contributions of steric and electrostatic forces to the interactions of a particle approaching such a gradient over both the swollen and the collapsed regions.^26^ However, this does not permit an inference of the composition of the gradient at a certain position. To establish a correlation between the composition of the films, the pH-dependent swelling, and other properties, we investigate the swelling and the monomer distribution in a series of sequentially grafted polymer bilayers, where both layers are homogeneously polymerized over the surface.

SI-PGP is an attractive preparation method due to its simplicity. In contrast to many controlled radical polymerization methods, it allows grafting onto almost any organic surface without the need for initiators or potentially toxic catalysts or ligands, or controlled atmospheres, and the polymer is easily formed in patterns^33,34^ or onto different sample geometries,^32^ with up to a 1000-fold reduction in the amount of used materials, in comparison to controlled radical polymerization methods.^30^ SI-PGP can also be used to provide a surface with initiators.^35^ The mechanisms involved in SI-PGP are not fully understood and are probably different depending on the photosensitizer, and aspects of the SI-PGP mechanism have been studied in different photosensitive monomer systems. Originally, Li et al.^36^ found that styrene monomers could act as photosensitizers, where photon adsorption leads to the formation of biradicals, which could initiate a free-radical polymerization reaction. Upon abstraction of a hydrogen radical from an organic substrate, these biradicals also formed surface radical sites for subsequent free-radical surface-initiated polymerization. Later, Wang et al. demonstrated that also acrylic monomers were amenable to self-initiated polymerization and grafting, proposing in a similar manner that the self-initiation mechanism occurs via excitation of the monomer to a triplet state in equilibrium with a biradical form of the vinyl group, with sufficient energy to abstract hydrogen from an organic substrate and initiate the grafting.^28^ Despite the simple and widely applicable implementation of the SI-PGP method, it is still not widespread, but the literature demonstrates that it is used for solving polymer coating problems in many different applications.^37−40^

X-ray reflectometry (XRR) and neutron reflectometry (NR) are commonly used tools for investigating submicron-thickness film structures.^41,42^ Via fitting of structural model representations to reflectivity data, properties such as volume fractions and polymer chain segment density distributions can be inferred. Whereas X-ray contrast is provided via electron density, neutrons interact with the nuclei of the sample. This allows the labeling of a molecule or layer by isotopic substitution,^43^ and the differences in neutron scattering lengths of H and D isotopes are used extensively for contrast enhancement in soft matter studies by NR.^44,45^ A fundamental problem in the study of copolymers is to distinguish the distributions of the monomers in a composite layer, and we use H → D substitution of one of the monomers to distinguish the distribution of this monomer in an otherwise protonated polymer layer. Ellipsometry is also commonly used for investigating thin film structures and interfaces.^46,47^ Spectroscopic ellipsometry allows more precise models, or modeling of more complex layers, by measuring the wavelength dependence of the ellipsometric angles Ψ and Δ. For inhomogeneous layers, effective medium approximations are used to calculate the refractive index of the mixed phase, based on the volume fractions and optical parameters of the components.

In this work, the monomer distribution and the swelling of 12 samples with two sequentially SI-PGP-grafted polymers of varying compositions were studied. These were prepared by first grafting a deuterated poly(methacrylic acid) (pdMAA) layer (at four different grafting times), whereafter a poly(aminoethyl methacrylate) (pAEMA) layer (three different grafting times) was grafted. Weak polyelectrolytes were selected to facilitate control of the ionization via the pH. The optical properties and the thicknesses of the dry films were investigated by ellipsometry and X-ray reflectivity; the compositions of the samples were determined by neutron reflectometry to determine the distribution of the dMAA monomers in the film. The swelling of the films was monitored with spectroscopic ellipsometry in the pH range from 4.5 to 10.5.

## Materials and Methods

### Chemicals

**Figure 1:**
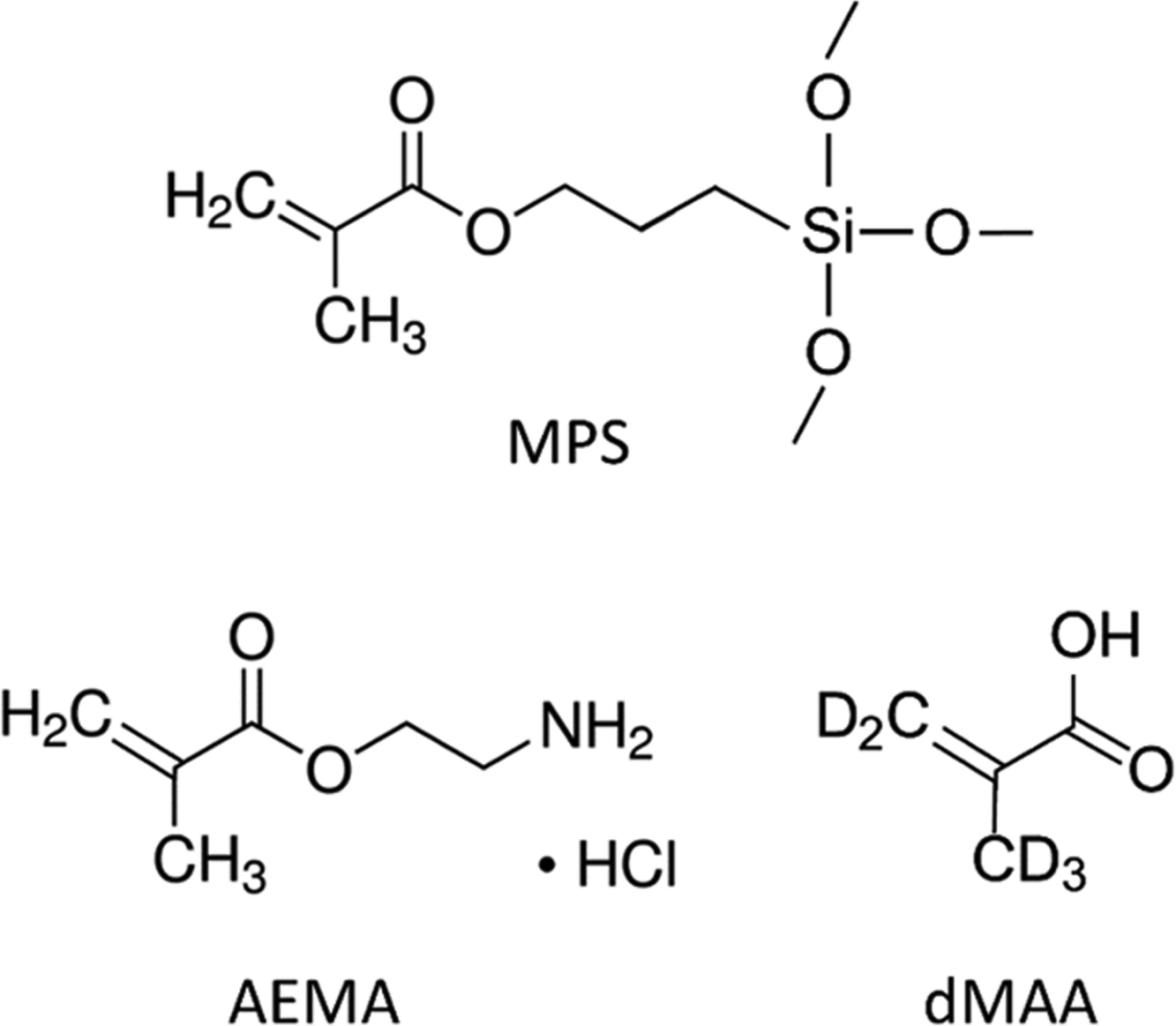
Structure of the silane [3-(methacryloyloxy)propyl]trimethoxysilane (MPS) and the used monomers, 2-aminoethyl methacrylate hydrochloride (AEMA) and deuterated methacrylic acid (dMAA).

Unless otherwise noted, water was taken from a Milli-Q source (Millipore) with 18.2 MΩ·cm resistivity, referred to as MQ water. The monomers (see Figure 1) 2-aminoethyl methacrylate hydrochloride (AEMA) and the deuterated methacrylic acid (dMAA) were obtained from Sigma-Aldrich (St. Louis, MO) and Polymer Source Inc. (Montreal, Canada), respectively. Ammonia, hydrogen peroxide, and sodium carbonate (AnalR Normapure) were obtained from VWR (Stockholm, Sweden), and ethanol was obtained from Solveco (Stockholm, Sweden). Tris(hydroxymethyl)aminomethane was purchased from SERVA Electrophoresis GmbH (Heidelberg, Germany). Glacial acetic acid was obtained from Merck (Darmstadt, Germany). [3-(Methacryloyloxy)propyl]trimethoxysilane (MPS), sodium acetate, sodium hydrogen phosphate, sodium dihydrogen phosphate, sodium bicarbonate, and hydrochloric acid were from Sigma-Aldrich.

### Sample Preparation

The samples used for this study consist of pdMAA and pAEMA layers, which were polymerized sequentially, in this order, using the SI-PGP method. The layers were deposited on 15 × 25 mm^2^ cut Si(100) 300 μm thick wafers with native oxide layers (Semiconductor Wafer, Taiwan).

An MPS layer was deposited onto the silicon surfaces to provide an organic layer to facilitate grafting. Before silanization, the samples were TL-1-cleaned (in a 5:1:1 mixture of water, 25% ammonia, and 30% H_2_O_2_ for 5 min at 85 °C, followed by rinsing with MQ water). The samples were then immersed into a solution of 20 mL water, 20 mL ethanol, 16 μL glacial acetic acid, and 160 μL MPS for 5 min. After drying under a stream of N_2_, the samples were baked in an oven for 10 min at 115 °C. Excess silanes were removed by sonication in ethanol for 1 min, and the samples were dried under a stream of N_2_.

For grafting, 0.1 μL/mm^2^ of a 0.25 M monomer solution was used (without any initiator). The wafers were suspended below a quartz plate using capillary forces of the monomer solution. The samples were then irradiated with UV light (Philips TUV PL-L, 18W, main emission peak at 254 nm) through the quartz plate. Three TUV PL-L lamps were placed side by side, 45 mm over the sample, in a box that fixes the lamp-sample distance to a predetermined distance. After illumination, the sample and the quartz plate were separated, and excess material was removed by ultrasonication in water for 5 min after each grafting. The second layer was deposited using a procedure identical to the first layer. A more detailed description of the grafting procedure can be found in previous works.^10^ The sample names reflect the polymerization times of the two layers: sample “S*ab*” indicates *a* min pdMAA polymerization and *b* min pAEMA polymerization.

### Sample Characterization

The layer thicknesses were monitored with X-ray reflectometry before and after the deposition of the second layer in a PANalytical EMPYREAN diffractometer equipped with a Cu Kα source operated at 45 kV and 40 mA. The incident optics was an X-ray mirror module equipped with a 1/32° divergence slit, and the reflected optics was a parallel plate collimator (0.27°). To determine the refractive index of the layers on each of the samples, optical measurements were carried out using a Mueller-matrix spectroscopic ellipsometer (MMSE) (J.A. Woollam, Lincoln, NE) in the wavelength range of 245–1700 nm at incident angles of 45, 55, and 65°. Both the X-ray and the Mueller-matrix ellipsometric measurements were performed under ambient conditions.

The swelling measurements were performed using an imaging nulling spectroscopic ellipsometer (EP3-SE, NanoFilm (now Accurion), Göttingen, Germany) equipped with a cell for liquid measurements, using 43 wavelengths between 350 and 930 nm at 60° angle of incidence, using two-zone averaging. For controlling the pH during the measurements, four different types of 10 mM buffer solutions were used. Acetic acid and sodium acetate solutions were mixed to obtain buffers in the pH range of 4–6, disodium and monosodium phosphate in the range of 6–8, Tris(hydroxymethyl)aminomethane and hydrochloric acid in the range of 8–9, and sodium carbonate and bicarbonate solutions were used to obtain buffers in the pH range of 9–11. The pH values were set by mixing the two components at the given ratios and were not adjusted otherwise and were determined before the experiment.

The compositions of the samples were determined by neutron reflectometry measurements. The reflectograms were recorded at the time-of-flight reflectometer SURF^48^ (samples S33, S35, S43, S53, S63) and OFFSPEC^49^ (sample S34) (Rutherford-Appleton Laboratory (RAL), Didcot, U.K.) and the single wavelength reflectometer NREX^50^ (FRM2, MLZ, Munich, DE) (all other samples). The measurements at SURF were carried out at three angles (0.28, 0.54, and 1.2°) covering a *Q* range of 0.008–0.15 Å^–1^, and the slits were set to maintain a resolution of 4%. The measurements at OFFSPEC were recorded using two angles (0.5 and 2.0°) that allowed us to record data in the *Q* range between 0.009 and 0.15 Å^–1^ at a resolution of 2.5%. At NREX, the angular range covered was between 0.14 and 2°, resulting in a *Q* range of 0.007–0.1 Å^–1^, and both upstream slits (separated by a distance of 2 m) were opened to 1 mm. All of the NR measurements were performed in a sealed chamber with Al foil windows while purging with dry N_2_ to reduce the humidity.

The prepared polymer films were visibly homogeneous over the sample areas. The lateral homogeneity was not explicitly investigated, but the conducted measurements cover relatively large areas. The spot size for XRR was 20 × 5.75 mm^2^ at the highest angle, for NR 25 × 23 mm^2^ at the highest angle in the case of the angle-dispersive instrument, and in the ToF measurement, the beam covered the whole sample. In ellipsometry, the spot sizes were on the order of 5 × 5 mm^2^.

### Modeling

The XRR data were fitted with GenX software^51^ using a two-layer model of a native oxide and one polymer layer with a bi-sigmoidal roughness, which was chosen to aid the modeling of the Muller-matrix ellipsometry data. Trivial models with sigmoidal roughness, which have plausible SiO_2_ thicknesses (i.e., greater than 10 Å), did not result in good fits to the data (see Figures S2 and S4 in the Supporting Information, from which it is clear that sigmoidal models reproduce the thicknesses but not the interfacial roughnesses of the layers). This is true for both the data of the pdMAA layer alone and the copolymer layer, suggesting that this is related to the interfacial structure at the polymer/air interface but not to the mixing of the two polymers. The bi-sigmoidal interface model allows the use of additional parameters to describe the interfacial structure. Further explanation of the bi-sigmoidal roughness and its parameters can be found in the Supporting Information. Due to the weak X-ray contrast between the two polymers, both the dMAA layer and the mixed layer were fitted by fixing the scattering length to that of dMAA (1.98 × 10^–3^ Å) and just varying the density of the films. The X-ray reflectograms recorded after the deposition of the second layer and the neutron reflectograms were fitted simultaneously, constraining the native oxide parameters to be identical. Since UV irradiation of Si surfaces can modify the native oxide layers,^52^ the parameters describing the oxide layer were fit independently for the samples having only the pdMAA layers. To avoid unphysical parameters arising due to the thickness values being smaller than the roughness values, the roughnesses on both sides of the SiO_2_ layer were constrained to be equal.^53^ The X-ray scattering length densities (xSLDs; we will refer to neutron scattering length densities as nSLDs) of the native oxide layer and the substrate were fixed to 20.06 × 10^–6^ and 18.8 × 10^–6^ Å^–2^, respectively. The fits were optimized using the logbars figure of merit (FoM), and the displayed error corresponds to a 5% increase in the FoM.

To evaluate the Mueller-matrix ellipsometry data, CompleteEASE software (J.A. Woollam Co.) was used. A model consisting of a native oxide layer and a Cauchy layer on top of the Si substrate was fitted to the measured data. A two-term Cauchy layer was used, where the refractive index is *n*(λ) *= A* + *B*/λ^2^. Since for thin layers, the refractive index and the thickness parameters of the film are correlated, the values for the thicknesses of the SiO_2_ and the polymer layers were taken from the XRR measurements and kept constant throughout the fitting.

From the ellipsometry data on the hydrated films, the polymer thickness and volume fraction parameters were calculated by modeling the sample with a SiO_2_ layer and a mixed polymer and water layer using EP4 View software (Nanofilm, Germany). The SiO_2_ thickness parameter was taken from the XRR measurements, and the Cauchy parameters of the polymer were taken from the dry ellipsometry measurements. To calculate the refractive index of the mixed layer containing polymer and water, a Bruggeman effective medium approximation was used.^54^ The resulting pH-dependent thickness and volume fraction curves were normalized to their maximum values and then simultaneously fitted with sigmoidal models. Samples showing one transition from a collapsed to a swollen state (“S”-type curves) are fitted with one sigmoidal curve, while samples with two transitions (“U”-type curves) are fitted with two sigmoidal curves. The maximum values for the sigmoidal curves were fixed to the greatest measured value.

The neutron reflectometry data were analyzed with the program GenX using a model consisting of a SiO_2_ layer and the polymer split into two layers. The contrast difference provided by the deuteration of one polymer component makes it meaningful to divide the polymer into more than one layer to reveal stratification within the film, which is not possible in the case of XRR, due to the low X-ray contrast between the polymer components. The X-ray reflectograms recorded after the deposition of the second layer and the neutron reflectograms were fitted simultaneously, constraining the native oxide parameters to be identical. To avoid unphysical parameters arising due to the thickness values being smaller than the roughness values, the roughnesses on both sides of the SiO_2_ layer were constrained to be equal,^53^ while the nSLD values were fixed to 3.5 × 10^–6^ and 2.07 × 10^–6^ Å^–2^ for the SiO_2_ layer and the Si substrate, respectively. During fitting, the thickness of the SiO_2_ layer was confined between 10 and 35 Å and the roughness values were limited to 0–35 Å. For the deuterated dMAA, we used an nSLD of 5.53 × 10^–6^ Å^–2^, and for the protonated AEMA, we used an nSLD of 0.92 × 10^–6^ Å^–2^. The nSLD values of the polymer layers were confined between these two values. The nSLDs of the monomers were calculated using the scattering lengths from ref (55) and number densities calculated from the mass densities, taking into account the isotope substitution. The mass density of dMAA was calculated from the MAA density^56^ and that for AEMA obtained using an online density calculator.^57,58^ The fits were optimized using the logbars figure of merit (FoM), and the displayed error corresponds to a 5% increase in the FoM.

## Results and Discussion

### Ellipsometry on Dry Films

The grafting times for the different samples, and the optical parameters resulting from the fitting of the Mueller-matrix ellipsometry data obtained on the dry films, are displayed in Table 1. There is no systematic variation in either of the obtained optical parameters with the grafting times, and excluding sample S44 (for reasons explained further down), the averaged Cauchy parameters are 1.484 ± 0.016 and 80.6 ± 2.0 × 10^–4^ nm^–2^. For many of the samples, the refractive indices fall between reported values of 1.537 for pAEMA^59^ and 1.475 for pMAA,^60^ but we note that samples S45, S54, and S55 have refractive indices lower than this range and also much lower than the average value. We attribute this to the large water content in the layer under ambient conditions.^61,62^

**Table 1:** Grafting Times for the Two Monomers, Thicknesses Derived from the Simultaneous XRR and NR Fitting, and Optical Constants of the Cauchy Model, Determined by Fitting of Mueller-Matrix Ellipsometry Data Obtained on Dry Samples (RIUs, Refractive Index Units)

sample	*t*_dMAA_ (min)	*t*_AEMA_ (min)	*d*_SiO_2__ (Å)	*d*_copolymer_ (Å)	*A* (RIU)	*B* (10^–4^ nm^–2^)
S33	3	3	16 ± 6	168.9 ± 1.9	1.524	76.7
S34	3	4	15 ± 3	195.9 ± 2.0	1.550	71.4
S35	3	5	16 ± 3	183.0 ± 1.6	1.493	82.0
S43	4	3	16 ± 7	217 ± 3	1.521	79.5
S45	4	5	16 ± 6	214 ± 7	1.426	89.6
S53	5	3	11 ± 9	184.7 ± 1.9	1.527	76.5
S54	5	4	35 ± 6	186 ± 4	1.350	94.8
S55	5	5	30.2 ± 1.2	176.6 ± 0.8	1.400	95.1
S63	6	3	17 ± 3	162.2 ± 1.9	1.489	85.9
S64	6	4	22 ± 5	188 ± 3	1.451	85.0
S65	6	5	24.3 ± 2.0	176.3 ± 0.8	1.473	84.0

### X-ray Reflectivity

X-ray reflectivity measurements were used to determine the dry thicknesses of the polymer layers after each of the two polymerization steps. Reflectivity profiles for all samples are displayed in Figures S1 and S3 (see the Supporting Information), and the parameters of the models are summarized in Table S1 for the first polymer layer and in Table S2 for the films after the second grafting onto the samples. For the fitting of the XRR data, a three-layer model was used, the first layer representing the native oxide and the other two the polymer layer, with the top layer thickness constrained to 0 Å, as described for the bi-sigmoidal profile in the previous section and in the Supporting Information. The results show that the differences in the thicknesses of the dMAA layers are small. The calculated average thickness of the initial dMAA layer weighted by the errors is 74.0 ± 1.5 Å, and the maximum deviation from the mean is 13.2 ± 1.7 Å. This similarity suggests that the samples are only significantly different in the amount of AEMA deposited. The average thicknesses of the resulting copolymer layers are 178 ± 10, 192 ± 3, and 177.3 ± 1.9 Å for 3, 4, and 5 min of pAEMA grafting, respectively. Note that the X-ray contrast between the two polymers is minimal, and it is not possible to distinguish the two polymers in the copolymer layers (xSLDs for AEMA and MMA are 9.3 × 10^–6^ and 9.1 × 10^–6^ Å^–2^, respectively). For the purpose of the following discussion, the samples with the same AEMA grafting times are considered as replicates of each other.

### Neutron Reflectivity

**Figure 2:**
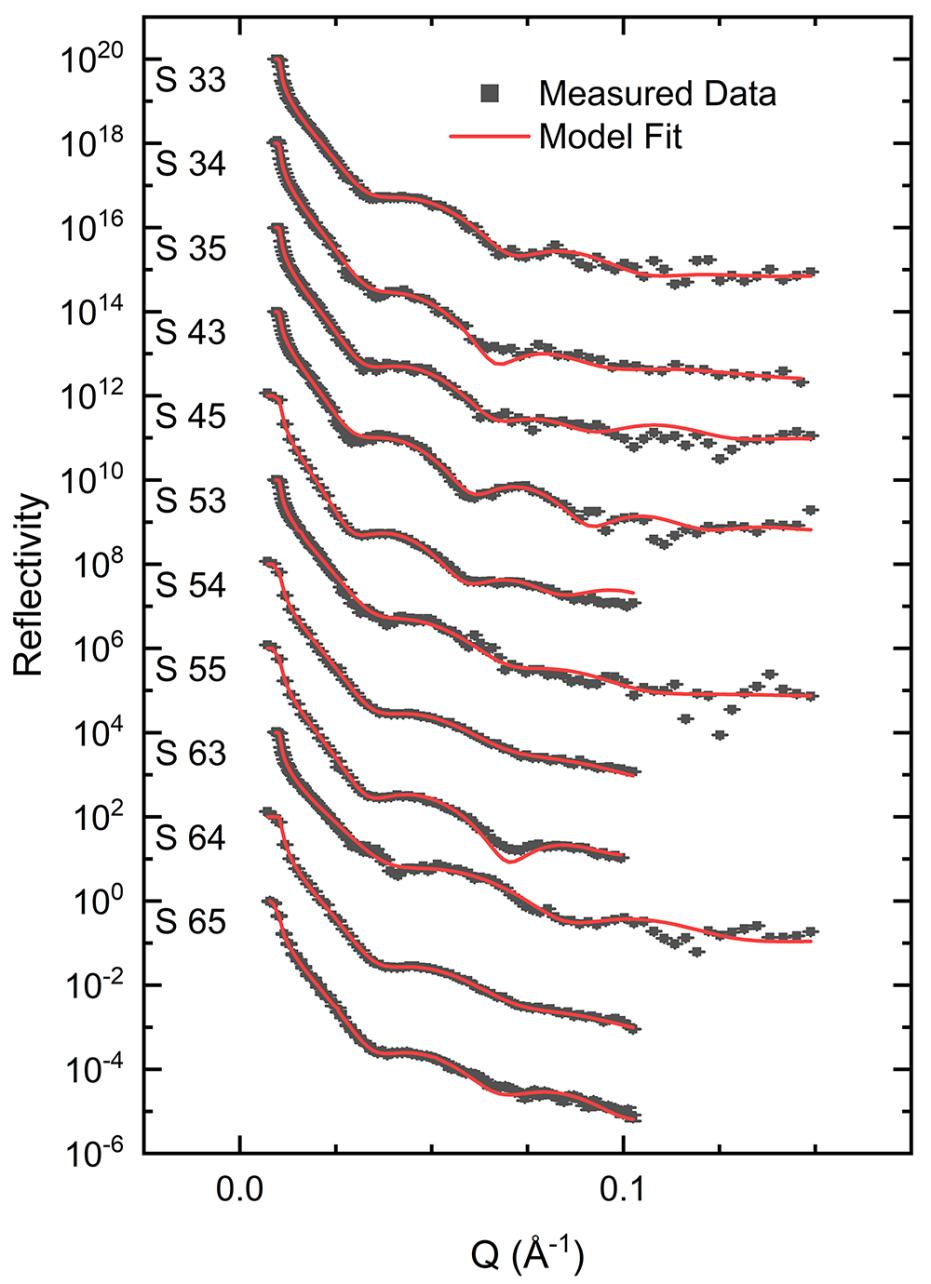
Neutron reflectivity data obtained on the copolymer layers in the dry state (black) and the corresponding model fits to the data (red). Data set S65 is correctly positioned relative to the vertical axis. Subsequent data sets have been scaled ×100 relative to the previous data set for clarity.

**Figure 3:**
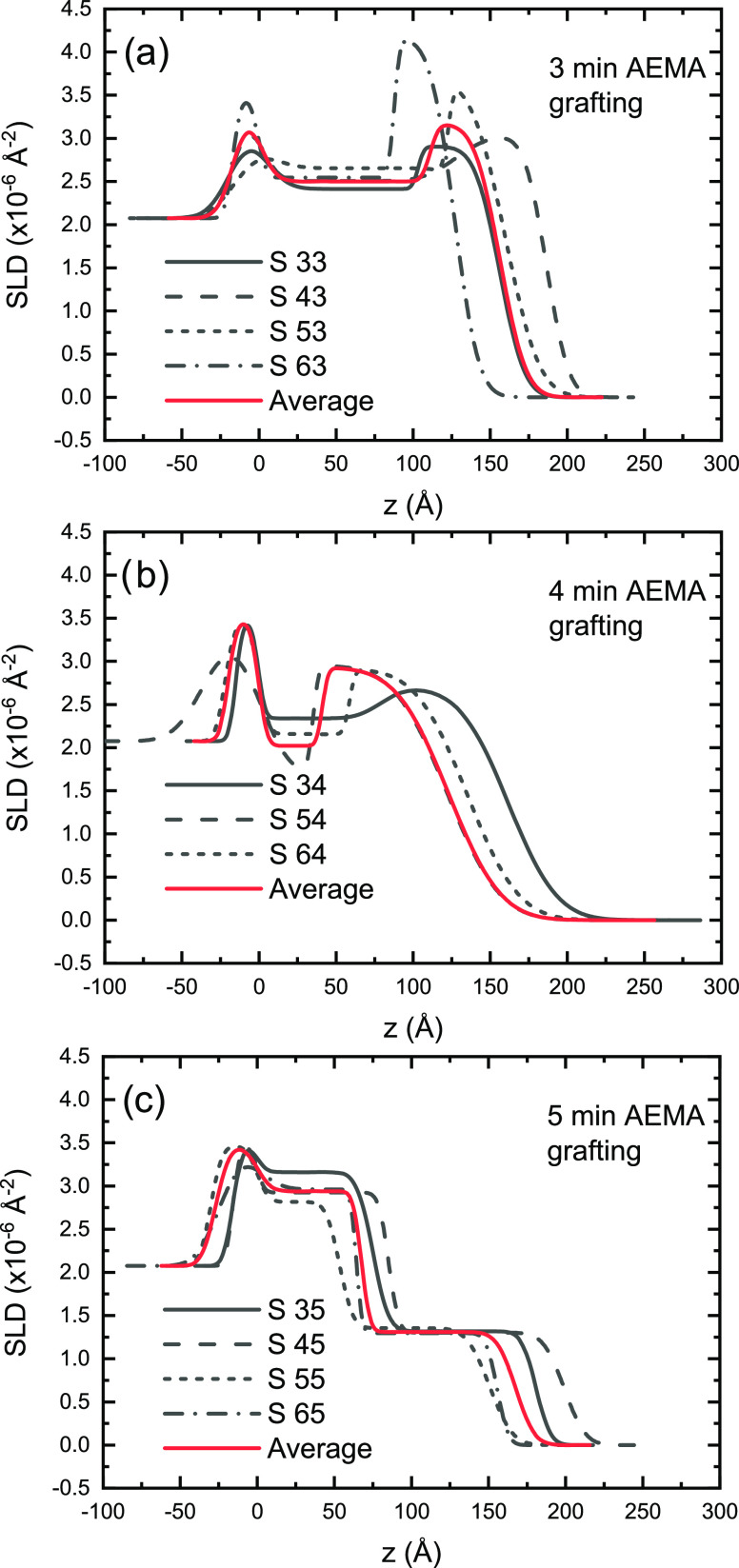
Neutron SLD profiles obtained from modeling of the reflectivity data. Black curves are calculated from individual samples, and red curves are averages for samples with the same AEMA grafting times: (a) 3 min, (b) 4 min, and (c) 5 min.

Neutron reflectivity measurements were conducted on the dry samples to distinguish the distributions of the two monomer types within the copolymer layers. The results of the NR measurements are shown in Figure 2. The polymer is modeled as a two-layer structure, and the parameters obtained from fitting this model to the data are shown in Table 2, with the corresponding calculated reflectivity profiles included with the data in Figure 2. The fit results for sample S44 suggest incomplete grafting of the pAEMA layer, and this sample is thus omitted from the further analysis and the discussion of the stratified polymer layer. The neutron SLD profiles obtained from the modeling are presented in Figure 3.

**Table 2:** Parameters of the NR Model Fits, Showing Layer Thicknesses (*d*), Neutron Scattering Length Densities (nSLDs), and Interfacial Roughnesses (σ)^a^

sample	*d*_top_ (Å)	nSLD_top_ (10^–6^ Å^–2^)	σ_top_ (Å)	*d*_bottom_ (Å)	nSLD_bottom_ (10^–6^ Å^–2^)	σ_bottom_ (Å)	*d*_SiO_2__ (Å)	σ_SiO_2__ (Å)	FoM	*d*_TOT_ (Å)
S33	53 ± 3	2.91 ± 0.05	12.7 ± 1.3	103 ± 3	2.41 ± 0.03	3 ± 12	16 ± 6	12.6 ± 1.6	0.87	156 ± 4
S34	83 ± 4	2.70 ± 0.06	25 ± 2	80 ± 4	2.34 ± 0.06	13 ± 13	15 ± 3	3.6 ± 0.8	1.24	162 ± 6
S35	106 ± 3	1.32 ± 0.09	7 ± 4	75 ± 2	3.16 ± 0.04	8 ± 4	16 ± 3	4.9 ± 0.7	0.97	181 ± 4
S43	59 ± 3	3.04 ± 0.09	11.4 ± 1.6	127 ± 3	2.50 ± 0.06	17 ±14	16 ± 7	9 ± 3	1.17	186 ± 5
S44	61.2 ± 0.4	5.53 ± 0.01	27.3 ± 0.2	24.6 ± 0.3	0.75 ± 0.04	31.8 ± 0.3	30 ± 6	11 ± 3	1.04	85.7 ± 0.5
S45	113.9 ± 1.5	1.30 ± 0.02	11 ± 2	85 ± 2	2.93 ± 0.02	5 ± 2	16 ± 6	3.7 ± 1.8	2.40	199 ± 3
S53	37 ± 3	3.70 ± 0.14	17.4 ± 1.8	123 ± 7	2.65 ± 0.05	3 ± 8	11 ± 9	14.6 ± 1.8	1.19	160 ± 8
S54	87.0 ± 1.1	2.95 ± 0.02	27.1 ± 0.4	35.0 ± 0.5	1.76 ± 0.03	3.0 ± 1.3	35 ± 6	16 ± 3	1.28	122.0 ± 1.2
S55	97.3 ± 1.7	1.35 ± 0.03	12 ± 2	53 ± 2	2.82 ± 0.03	7 ± 4	30.2 ± 1.2	5.8 ± 0.4	2.86	151 ± 3
S63	39 ± 2	4.16 ± 0.11	12.4 ± 1.7	88 ± 5	2.54 ± 0.05	3 ± 7	17 ± 3	4.2 ± 1.1	1.22	127 ± 6
S64	76.5 ± 1.1	2.91 ± 0.02	26.2 ± 0.4	58.7 ± 0.8	2.16 ± 0.02	3 ± 2	22 ± 5	5.0 ± 1.8	1.57	135.2 ± 1.4
S65	91.4 ± 1.2	1.30 ± 0.03	6 ± 3	64.2 ± 1.7	2.96 ± 0.02	3.0 ± 1.6	24.3 ± 2.0	12.1 ± 0.6	1.56	156 ± 2

a Indices top and bottom refer to the two layers used in the modeling of the polymer; these are not necessarily the pdMAA and the pAEMA layers. For clarity, also the total thickness is presented in the last column.

It is clear from both the XRR and the NR results that the profiles from samples with the same pAEMA grafting times are very similar, irrespective of the pdMAA grafting time, and hence also the averaged profiles from all samples with the same pAEMA grafting times have been calculated using error-weighted averages and are included in Figure 3, with the resulting averaged layer parameters displayed in Table 3. In the rest of the discussion, the average properties of all of the samples with the same pAEMA deposition time are considered. The variability between the samples can be estimated from the errors of the averaged values displayed in Table 3. This amounts to ca 20% for the largest variation (8 Å thickness variation for a 41 Å layer) but is in most cases around 10%. The differences between the total layer thickness values determined from the X-ray and neutron reflectometry results arise from the different measurement conditions, where the former were acquired under ambient conditions and the latter under dry N_2_ purging. Hydrophilic polymer films are hydrated and swell due to the vapor content under ambient conditions,^61,62^ resulting in generally larger XRR than NR thicknesses, in this case. The contrast between water (nominal xSLD 9.45 × 10^–6^ Å^–2^) and AEMA (xSLD 9.3 × 10^–6^ Å^–2^) is too small for X-rays to allow these components to be distinguished, and hence, the swelling caused by water vapor is considered as an increase in thickness.

**Table 3:** Parameters of the NR Model Fits, Averaged over the dMAA Grafting Times

sample	*d*_top_ (Å)	nSLD_top_(10^–6^ Å^–2^)	σ_top_ (Å)	*d*_bottom_ (Å)	nSLD_bottom_ (10^–6^ Å^–2^)	σ_bottom_ (Å)	*d*_SiO_2__ (Å)	σ_SiO_2__ (Å)
3 min average	45 ± 5	3.2 ± 0.3	13.2 ± 1.2	111 ± 8	2.50 ± 0.05	4 ± 2	16.0 ± 0.9	9 ± 3
4 min average	82 ± 4	2.93 ± 0.03	26.7 ± 0.3	41 ± 8	2.0 ± 0.2	3.1 ± 0.6	20 ± 5	4.4 ± 1.9
5 min average	100 ± 5	1.31 ± 0.01	9.7 ± 1.4	67 ± 6	2.94 ± 0.05	4.4 ± 1.0	27 ± 3	7.1 ± 1.6

**Figure 4:**
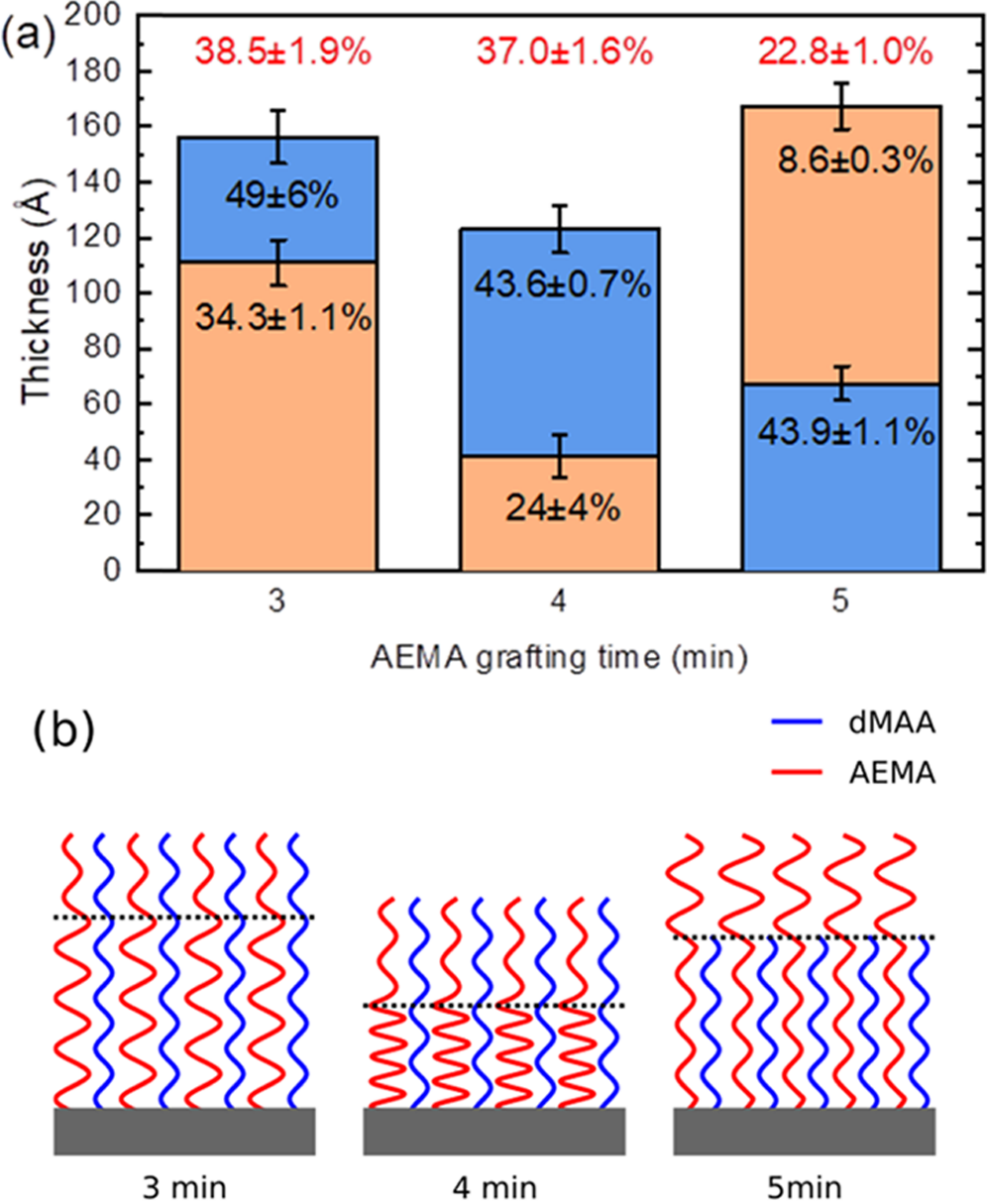
(a) Polymer bilayer model showing the two distinct layers obtained from fitting of the neutron reflectivity data. The percentages indicate the pdMAA fractions in the layers (red numbers are average pdMAA fractions for the entire film). The blue color indicates regions of a near 1:1 ratio composition, and the amber color indicates AEMA-rich layers. (b) Schematic illustration of the evolution of the film structure, visualizing the rearrangement of AEMA upon increasing grafting times. The dotted lines indicate the boundaries between the layers shown in panel (a). Note that while the polymers are depicted as unbranched chains, this is merely to illustrate the relative amounts of the two components but not intended to reflect the actual polymer structure.

The dMAA content of the films calculated from modeling of the neutron data is shown in Figure 4a. Note that the two layers in the model do not necessarily reflect the (nominal or actual) thicknesses of the sequentially deposited layers but is dependent on the distribution of the deuterated monomer in the film, as inferred from the contrast difference between the protonated (AEMA) and deuterated (dMAA) monomers and also illustrated schematically in Figure 4b. The absence of layering, which directly reflects the sequence of polymer deposition, shows that the second (protonated) layer is not merely grafted from (or onto) the top of the preexisting first (deuterated) layer during deposition. The obtained structure can be explained within a polymer growth model with independent grafting of the two polymers from solution to the substrate. The lack of a low-nSLD layer on top of the dMAA film and the abundance of protonated material (AEMA) near the bottom of the film for the first two sets (3 and 4 min AEMA polymerization, respectively) suggest that the growth of the p(AEMA) polymer proceeds from the surface of the substrate, not from the top of the previously deposited layer. This has some similarities to the “grafting-through” model,^63^ with polymerization in solution and subsequent grafting of oligomerized or polymerized fragments to the substrate and also as suggested for SI-PGP already by Wang and Brown.^28^ The decrease in dMAA in the bottom layer between 3 and 4 min does not primarily reflect the removal of dMAA from this layer but the addition of AEMA, resulting in a lower MAA fraction. The reaction is initiated by short-wavelength UV radiation that not only creates radicals, which sustain the polymerization reaction, but also degrades the grafted layer, in accordance with previous observations.^29^ The decrease in the high-nSLD pdMAA fraction in the entire films (red numbers in Figure 4a) is a consequence of both this process and dilution by the increasing amount of protonated pAEMA. The apparent reversal of the structure for the thickest layer (5 min AEMA grafting) is attributed to the change in relative thicknesses of the two contributing polymers. For short AEMA grafting times, the length of the formed pAEMA chains does not exceed the thickness of the existing pdMAA layer, and the top layer (in the two-layer model) has a near 1:1 composition, possibly a result of ion pairing between the polymers. When the sample is dried, this top layer collapses onto the bottom layer, burying the low-nSLD material on the bottom. With increasing AEMA grafting time, this effect is less dominant for the sample with a 4 min AEMA grafting time. However, as the thickness of the pAEMA layer exceeds that of the pdMAA layer in the 5 min AEMA sample, the collapse is inverted and the top layer is dominated by AEMA extending beyond, and upon collapse, covering the pdMAA layer. Thus, since AEMA has been expelled from the bottom layer, the dMAA volume fraction in this layer increases without changing the absolute amount of dMAA much. The inversion is driven by the electrostatic repulsion of the excess of positive charges in the bottom layer, eventually expelling the fraction of segments that are not required to maintain charge balance to the top, as the pAEMA layer is growing.

Rearrangement of polymer chains, similar to that shown in Figure 4, with the reversal of top and bottom layers, has been observed in mixed polyelectrolyte brushes in response to changes in pH.^64,65^ While these systems do not immediately reflect responses to changes in molecular weight or composition, as we see for prolonged grafting, they represent similar phenomena, in that they are structural rearrangements driven by changes in the ratio of cations to anions in the brushes. Similar phenomena have also been observed in response to wetting^66^ and solubility changes.^67^

Fitting the neutron reflectivity data using a one-layer model for the polymer results in worse fits, with an average increase in the FoM of 110%, compared to those obtained using the two-layer model. We note that the differences are far greater than the 5% increase in FoM that was used for error estimation for all samples except S34. The single-layer fits and a detailed comparison of the FoMs are presented in the Supporting Information (Table S4 and Figures S6 and S7). That a one-layer model is still possible to use with reasonable results supports the view that the second layer is not grafted on top of the first but that the second layer is intermixed with the first. In the one-layer model, there is a monotonous decrease in the polymer layer nSLD with increasing pAEMA content, as expected (considering the nSLD profiles for the average of samples with the same AEMA grafting times, as above). The resulting average layer thicknesses and the total dMAA contents in the films are similar in the one- and two-layer models (see Table S3). The one-layer model also retains S44 as an outlier. However, since the two-layer model generates overall better fits and reveals plausible internal structuring of the polymer layer, in that it accounts for the grafting depth of the pAEMA layer and also predicts the expected degradation of the pdMAA layer; we argue that it has superior explanatory power, and we consider that its use is justified.

### Spectroscopic Ellipsometry on Wet Films

**Figure 5:**
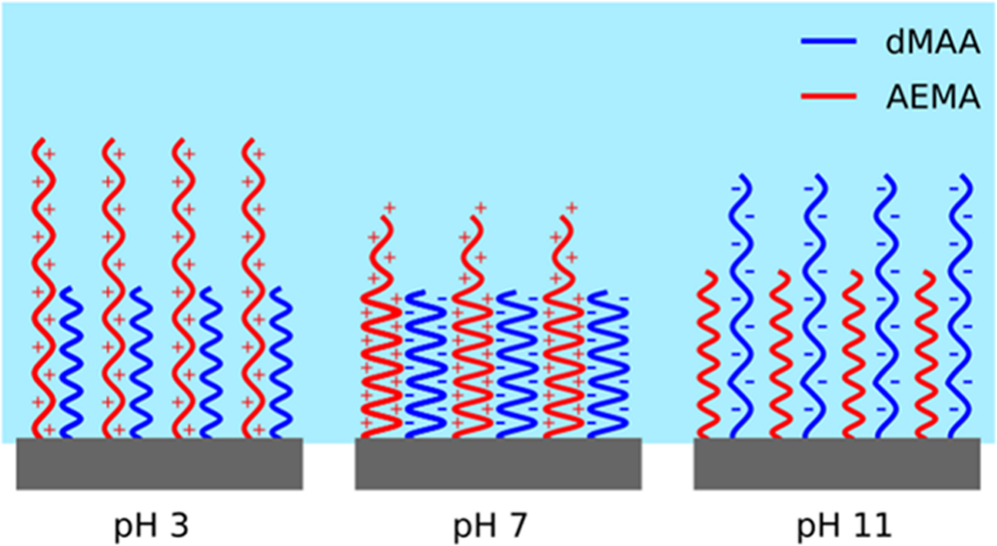
Schematic structure of the hydrated polymer layers at different pH values, visualizing the rearrangement of the respective components upon variations in pH. Note that while the polymers are depicted as unbranched chains, this is merely to illustrate the relative amounts and the expansion or contraction of the two components but not intended to reflect the actual polymer structure.

By spectroscopic ellipsometry, we were able to monitor the evolution of the thickness and hydration of the copolymer films with changing pH except for films S54 and S55 (see the comment on these in the following). The films are in a collapsed state at and around neutral pH and swell considerably at high and/or low pH due to excess ionization and subsequent electrostatic expansion of the polymer in pH regions where the anionic and cationic residues are not neutralizing each other. As is clear from Figure 4a, the compositions of the films are such that AEMA dominates over MAA for all AEMA grafting times. However, under wet conditions, the overall behavior is that of a polyampholyte, where the overweight of AEMA residues does not prevent swelling of the polyanionic pdMAA chains. The resulting hydrated structures are schematically represented in Figure 5. Qualitatively, the behaviors are similar at a given pH for films with different AEMA grafting times. At low pH, the swelling is caused by neutralization of the MAA residues, leading to low solubility and collapse of the pdMAA and expansion of the pAEMA chains due to charge–charge repulsion. Similarly, at high pH, deprotonation of the AEMA residues reduces the polarity of the pAEMA segments, and swelling is caused by expansion of the now deprotonated and anionic pdMAA chains. In either case, partial neutralization by charge–charge interaction between the polymers is possible, though the extent of any such interaction is unknown to us since we have no information about the wet structure of these polymer layers, and important parameters such as the chain segment density distributions or local variations in dielectric functions^68^ remain unknown. At intermediate pH, where both MAA and AEMA residues are charged, compaction of the polymer due to extensive charge–charge interaction is expected. Considering the obtained hydrated thicknesses of the polymer layers in this region (see, e.g., Figure S8), and comparing them to the dry thicknesses (Table 1), this indicates some swelling, even considering the uncertainty of the ellipsometric thickness data of the hydrated films. This is attributed to repulsion between protonated excess AEMA residues, which are in majority for all AEMA grafting times. In this AEMA-rich environment, there are ample opportunities for AEMA residues to neutralize the dMAA by forming chelates or inner salts.

**Figure 6:**
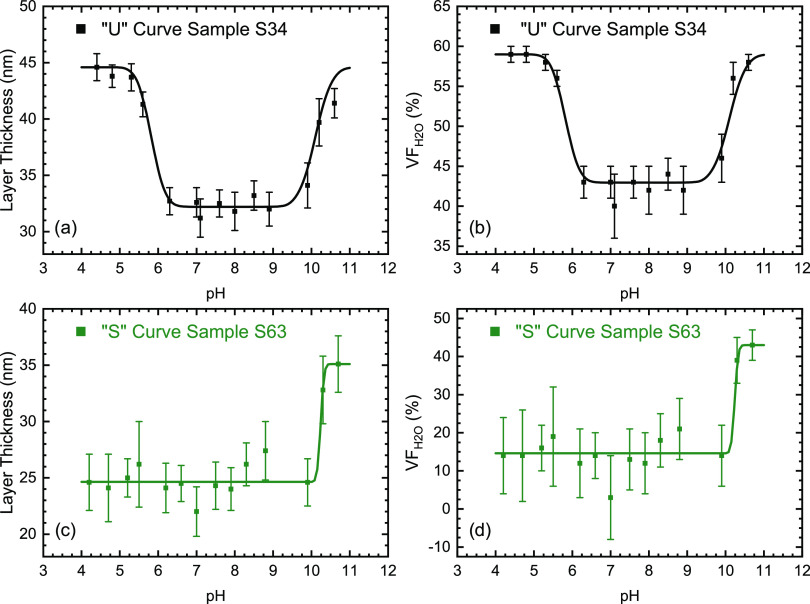
Example ellipsometry data showing layer thicknesses and water volume fractions and also illustrating the two types of swelling behaviors observed, with (sample S34, (a) and (b)) and without (sample S63, (c) and (d)) an observable lower swelling transition pH, respectively. A complete set of data, for all samples, is included in Figure S8 (Supporting Information).

From the swelling behavior of the films, the values of the transition pH at high and low pH, respectively, were monitored and are summarized in Table 4. Plotting the swelling versus pH, the expected behavior is a “U”-shaped curve with a transition at low pH where the carboxyl groups become protonated and a transition at high pH where the protonated amine groups become deprotonated. An example of this behavior is shown in Figure 6a,b. The details of the used models and the fits for the individual samples are presented in the Supporting Information (Figure S8). The location of the higher transition pH is constant over the composition range with an average value of 10.15 ± 0.04. This value is close to the p*K*_a_ of a protonated primary amine 10.6,^69^ while the p*K*_a_ of an AEMA monomer is 8.8^70^ and that of a pAEMA layer was found to be 7.6.^70^ We attribute this difference to the presence of negative charges in the copolymer film, creating an environment where many more AEMA residues are neutralized than would be the case in a pAEMA film and thus reducing charge regulation effects. The average lower transition pH is 5.3 ± 0.2. This value is higher than the reported p*K*_a_ value of the methacrylic acid monomer (4.65).^71^ The latter average does not take into consideration the curves that do not exhibit a low pH transition within the observed pH range, such as those in Figure 6c,d. Since the amplitudes of the transitions and the inflexion points of the sigmoidal curves are not independent, the amplitudes of the transitions were fixed to the maximum values in a given measurement. Due to the water content of the films under ambient conditions, with moisture absorbed from the air influencing the refractive indices, the changes in the hydrated thickness and water volume fraction values contain no information about the films. In the case of samples S54 and S55 where the refractive indices were greatly influenced by the water content, the model fits did not converge, as was the case with certain pH values for samples S45 and S64. We note that all four samples have Cauchy parameters *A* < 1.46. To investigate the thickness and volume fraction behaviors, a more precise determination of the Cauchy parameters of the actual polymers is needed. A typical procedure to achieve this would consist of vacuum drying of samples for 24 h before determining the refractive index;^72^ however, one could determine the precise water content of submerged samples by varying the contrast of water in a neutron reflectometry experiment and model the refractive index using ellipsometry data recorded in parallel. This method could lead to better results in swelling experiments.^25,26^

**Table 4:** Ellipsometry Data from the Wet Characterization, Showing dMAA Volume Fraction (*c*_dMAA_), Lower and Higher Transition pH (pH_–_ and pH_+_, respectively), and the Width of the Corresponding Transitions (σ_–_ and σ_+_)^a^

sample ID	*c*_dMAA_ (%)	pH_–_	σ_–_	pH_+_	σ_+_
S33	36.0 ± 0.6	4.83 ± 0.11	1.3 ± 0.2	10.11 ± 0.05	0.38 ± 0.08
S34	34.7 ± 1.0	5.82 ± 0.06	0.41 ± 0.08	10.10 ± 0.05	0.49 ± 0.09
S35	25.1 ± 1.2	5.93 ± 0.13	0.63 ± 0.17	10.01 ± 0.05	0.34 ± 0.10
S43	38.0 ± 1.1	4.95 ± 0.07	0.80 ± 0.11	10.05 ± 0.04	0.52 ± 0.08
S45	23.4 ± 0.4	n.a.	n.a.	10.42 ± 20	0.09 ± 65
S53	42.9 ± 1.2	4.14 ± 0.20	1.4 ± 0.5	10.01 ± 0.05	0.45 ± 0.10
S54	36.1 ± 0.3	n.a.	n.a.	n.a.	n.a.
S55	20.7 ± 0.6	n.a.	n.a.	n.a.	n.a.
S63	46.0 ± 1.2	n.a.	n.a.	10.23 ± 400	0.09 ± 900
S64	36.1 ± 0.3	3.7 ± 0.2	1.0 ± 0.4	10.28 ± 0.02	0.44 ± 0.07
S65	23.1 ± 0.5	4.2 ± 0.3	1.6 ± 0.6	10.13 ± 0.05	0.52 ± 0.09

a n.a., not applicable, meaning that no transition was observed. The large error values in the case of samples S45 and S63 originate from the large correlation between the position of the inflexion point and the width of the transition.

**Figure 7:**
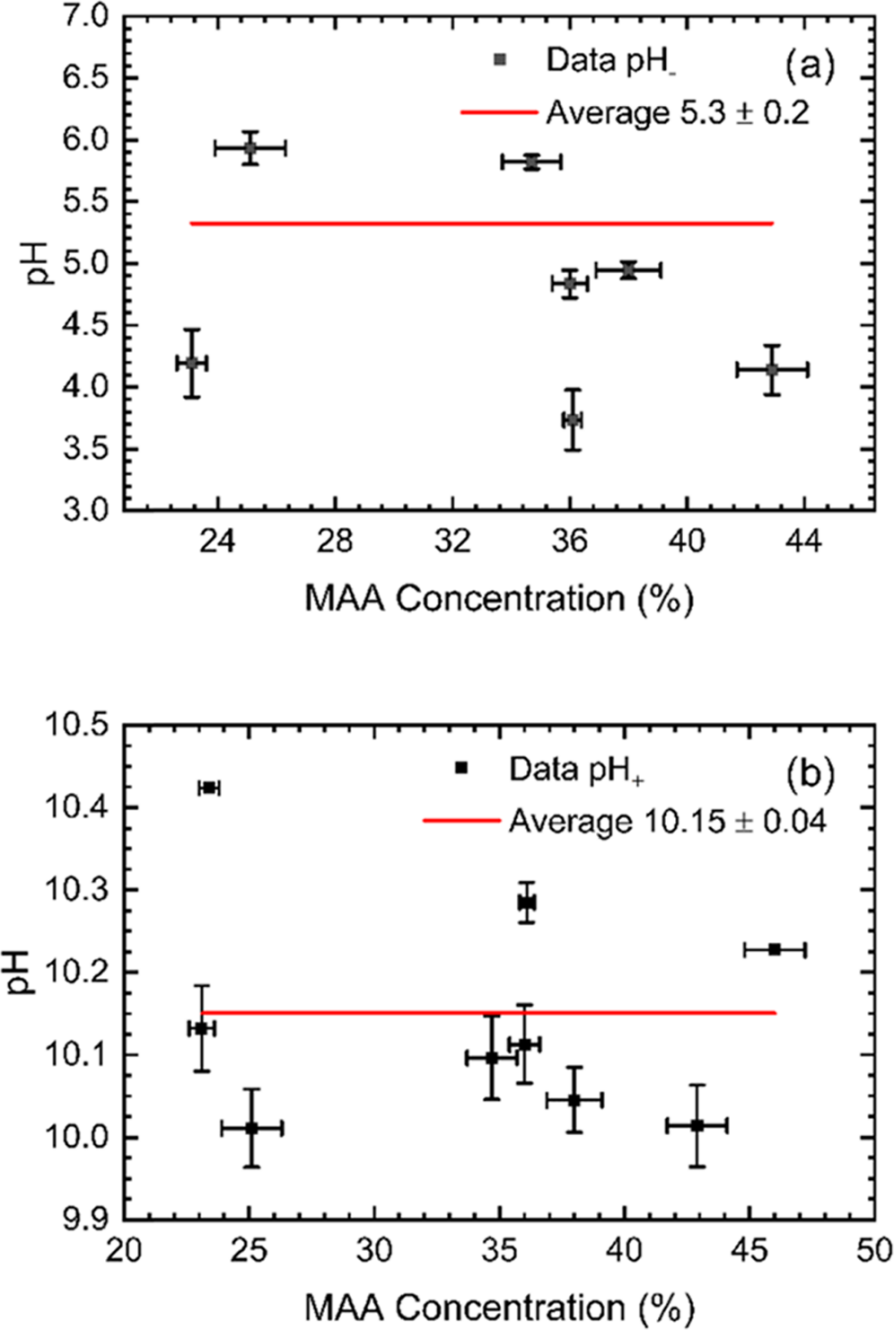
Summary of the analysis of the ellipsometric data. The MAA concentrations for each sample were calculated from the fitting of the NR data. (a) Lower transition pH and (b) higher transition pH. The data without y error bars correspond to having error bars larger than 14. These large error values in the case of samples S45 and S63 originate from the large correlation between the position of the inflexion point and the width of the transition.

In the “U”-type swelling curves (Figures 6a,b and S8), the transitions at low pH are in most cases more diffuse than those at high pH, extending over a greater pH range, as illustrated in Figure 7. This is in agreement with previous studies, which demonstrate clear differences in the swelling between regions rich in cations and anions, respectively, with variations in pH. Changes in regions dominated by protonation and deprotonation of anions were continuous over a broader pH range,^25,26^ while transitions caused by (de)protonation of cations occurred over a narrower pH range. This is the behavior displayed in most of the swelling curves observed also in our case (Figure S8). In the case of the “S”-type swelling curves (Figure 6c,d; samples S45 and S63), we attribute the lack of the transitions at low pH to the narrow pH range of the measurements. These two samples do not otherwise deviate or represent outliers, compared to the other samples, and there is nothing to indicate that a qualitatively different behavior of these samples should be expected. However, asymmetric swelling can be a result of ion-specific interactions,^73^ and with different buffers for the used pH range, this source of differences cannot be excluded.

### Implications for the SI-PGP Method

Uncertainty about the exact mechanisms involved in SI-PGP limits advanced uses of this otherwise simple and robust polymerization method. The monomers excited by UV irradiation form radicals with enough energy to initiate free-radical polymerization. Wang et al. suggested that the self-initiation mechanism occurs via excitation of monomers with sufficient energy to abstract hydrogen from an organic substrate and to initiate the grafting.^28^ However, this does not exclude the possibility of the formation of radicals on the already existing polymer chains. Wang et al. also observed an initial acceleration in the grafting rate (or, to be accurate, of the grafting conversion) with time, suggesting that this depended on the grafting of either monomers or chains onto already grafted chains and that this was facilitated by the high solubility of chains and/or monomers with each other.^28^ Since this is an uncontrolled polymerization reaction where polymerization proceeds in the bulk, and where monomers, oligomers, or polymers are grafted, while possibly also cross-linking and branching of grafted chains occur, the resulting polymer is expected to be heterogeneous. However, our results are consistent with a model where the grafting of solution-polymerized chains to the substrate surface is a dominating process and has some similarities to the grafting-through process^63^ (see further comments below). Grafting to existing chains cannot be excluded, but the extent of this is not possible to estimate from our data.

Previous work on SI-PGP-prepared ampholytic sequentially grafted thickness gradients has demonstrated that their swelling and net surface charge can be manipulated by the solution pH and also that they form a region of strong fouling resistance that can be relocated via the pH. This demonstrates that sequential grafting to achieve a pseudo-zwitterionic-like polymer is a viable route and can be used to prepare practically useful pH-responsive polymers. However, since the continuous UV degradation of the initially grafted layer hinders the preparation of samples with an accurate predetermined composition via grafting of a second layer, this is a procedure with certain disadvantages. Thus, we propose that for the preparation of pseudo-zwitterionic or ampholytic polymer films via the SI-PGP process, the sample composition should be controlled in a more conventional manner, using the composition of the grafting solution and adjusting the result via its ionic strength and the pH. The inevitable UV degradation^29^ also leads to a reduction in polymerization rate with time, and in combination with strong UV absorption of the water and the monomers, both the exposure time and the thickness and concentration of the monomer solution limit the layer thicknesses that can be achieved. A possible way around this is to interrupt the polymerization before monomers are consumed and before rate decrease is significant and to restart the process. However, our finding that polymerization in a second grafting step proceeds mainly by grafting of chains directly to the surface, and not to the existing polymer layer, also raises questions about this procedure. Instead, this suggests that the grafting densities of such films can be improved by the application of a second (and perhaps even a third) grafting step to increase the grafting density in SI-PGP-prepared homopolymer systems.

The observed insensitivity to the dMAA grafting time on the resulting layer thicknesses means that the thickness of this layer reaches a plateau already at the shortest grafting times. The reason for this is unclear, but grafting rates vary between monomers,^28^ much depending on solubility, for ionic monomers also on net charge, solution salinity, and pH. For UV-initiated polymerization in general, film growth proceeds until some point where grafting is no longer dominating over degradation.^29^ For rapidly polymerizing monomers, the depletion of monomers due to consumption and subsequent monomer diffusion-limited growth would limit the polymerization rate before all monomers are consumed. Since polymerization proceeds in bulk, with subsequent grafting to the surface, it is also conceivable that the increased viscosity of the bulk phase renders the diffusion of chains to the surface increasingly difficult, hence preventing the grafting of chains to the surface even when propagation reactions continue in the bulk. On the other hand, large kinetic isotope effects usually observed for deuterated compounds^74^ would suggest that the polymerization of dMAA is slow, making it less likely to have reached a plateau after only a short time.

A characteristic feature of the grafting-through process is the occasional inclusion of surface-bound monomers into chains otherwise formed in a bulk free-radical polymerization reaction.^63,75^ This includes a “grafting-to” step where polymers or oligomers diffuse to the surface to react with a surface-bound monomer and thereafter grow via a “grafting-from” mechanism fed by monomers diffusing from the bulk. In this process, steric hindrance by the successively denser surface polymer layer results in self-limiting grafting, leading to thicknesses that are largely independent of the reaction conditions.^63,75^ A growth model where solution-polymerized chains are grafted to the surface would lead to similar thickness limitations, though there are certain observations suggesting that grafting through is not at work here. First, the possibility of growing a second layer, also via grafting of solution-polymerized chains to the substrate surface, is not consistent with a thickness-limiting polymer layer providing steric hindrance. Second, reports on SI-PGP preparation with self-limiting thicknesses but which do not use surface-bound polymerizable groups (but various organic layers)^29,30^ show that grafting through is at least not involved in all SI-PGP reactions. Further experiments to shed light on the relevance of a grafting-through mechanism—or of other aspects of the mechanism—would be of interest but are hindered by the limited availability of both the bulk polymerized chains (due to the very small liquid volumes used and the high viscosity after preparation) and the small amount of surface-grafted material (which cannot be increased via growth on particles, as is common for many other processes).

In the preparation of pseudo-zwitterionic coatings, such as copolymerization from anionic and cationic monomers or the formation of self-assembled monolayers, it is often observed that the surface composition is largely insensitive to variations in the solution composition over a wide range of mixing ratios of the two ionic components.^76,77^ This is caused by the electrostatic interaction of the two oppositely charged components in the solution during formation, pairing the ions before surface attachment. We note that ion pairing is also extensively exploited on a larger scale in the layer-by-layer method, where alternating anionic and cationic polymers are adsorbed to form polymer multilayers with controlled properties.^78^ It is probable that electrostatic interactions contribute to the organization of the film during the second grafting step in our case, but the data are not conclusive on this point. The analysis of the NR data yields a stratified polymer film, where each averaged composition in Figure 4 shows a region of near 1:1 ratio of dMAA and AEMA, in addition to a layer with an excess of AEMA. This can be explained by a neutralization of the dMAA-rich regions with added pAEMA due to charge–charge interactions and subsequent location of pAEMA where the dimensions of the grafted chains permit, i.e., near the substrate for short grafting times, and on top of the dMAA-rich region for longer grafting times. However, we are unable to verify either the sequence of events or the internal organization of the polymer film, beyond the distribution of the monomers. The shift in the p*K*_a_ of AEMA in the polymer, as compared to that of the free monomer, demonstrates that electrostatic interactions are present in the polymer film, but it is not clear to what extent these influence the resulting monomer distribution (or the final structure). In the two-layer model, there is a clear preference for grafting to the surface in the second polymerization step, and the overshooting pAEMA-rich layer for the longest AEMA grafting times, containing approximately 90% AEMA, shows that the growth is not limited to (or by) ion pairing of monomers or chains. Similarly, the presence of an AEMA-rich layer near the bottom for 3 and 4 min AEMA grafting times (Figure 4) shows that the grafting of chains to the substrate, as opposed to the existing chains, is not driven (only) by the electrostatic association of AEMA chains with dMAA. If this were the case, grafting would seize as the dMAA moieties were charge-compensated, but the AEMA-rich layers near the bottom indicate that this is not the case.

## Conclusions

We have investigated the structure and the pH-dependent swelling of a series of sequentially grafted polyelectrolyte layers, using the SI-PGP method to graft pAEMA layers on top of a series of pdMAA layers. The dry samples were investigated by ellipsometry, X-ray, and neutron reflectometry, and their swelling under different pH conditions was monitored with spectroscopic ellipsometry. The dry sample compositions suggest that the growth of the second polymer layer proceeds via grafting of solution-polymerized fragments to the surface through the vinyl groups of the silane layer and not as a continuation of the chains of the initial layers. In the dry state, the films are stratified, with a region of near 1:1 monomer composition formed after the second polymerization step, and excess monomers accumulated either beneath or above this layer. For short grafting times of the second layer, the excess AEMA residues are compressed beneath the initial layer, near the substrate. For longer grafting times, the second layer reaches a sufficient thickness to extend above the initial film, forming a reversed stratified polymer, with a layer of near 1:1 monomer composition at the bottom. The presence of layers with excess monomers of one type shows that ion pairing during polymerization is not critical for the formation of the films. The ellipsometry results show changes in the swollen thickness and water content on the film according to the protonation or deprotonation of the two types of ionizable residues. The transition pH for swelling due to charging of the AEMA monomer is significantly higher than the reported p*K*_a_ of the monomer of the homopolymer, an effect ascribed to charge regulation in the polyelectrolyte environment, confirming that electrostatic interactions are at work in the polymer. We observe that in the investigated polymer composition range, there are no significant differences in the pH dependence of these changes, in contrast to what is observed on sequentially grafted polymer thickness gradients. Thus, while providing a robust procedure with little sensitivity to fine changes in the monomer compositions, this also implies that much of the tunability observed in thickness gradients was lost. However, the obtained results suggest instead that grafting density in SI-PGP-prepared polymers could potentially be increased via repeated polymerization steps in the preparation of homopolymer films.

## Supporting Information Available

X-ray reflectivity data, explanation of the bi-sigmoidal roughness model, comparison of the one-layer and the two-layer models for fitting neutron reflectometry data, the model used for fitting the spectroscopic ellipsometry data, and thickness and water volume fractions by spectroscopic ellipsometry for all samples (PDF)
