# Supplementary material for: Structure and pH-Induced Swelling of Polymer Films Prepared from Sequentially Grafted Polyelectrolytes
Source: Langmuir. 2022 Jan 26;38(5):1725–37. doi: 10.1021/acs.langmuir.1c02784 (PMC8830213; doi:10.1021/acs.langmuir.1c02784)
Supplement: Supplementary file 1 — la1c02784_si_001.pdf [file la1c02784_si_001.pdf]

Supporting Information

Structure and pH-induced swelling of polymer films prepared from sequentially grafted polyelectrolytes

Béla Nagy[1], Mario Campana[2], Yury N. Khaydukov[3,4], Thomas Ederth[1,*]

[1] *Division of Biophysics and Bioengineering, Department of Physics, Chemistry and Biology, Linköping University, SE-581 83 Linköping, Sweden*

[2] *ISIS Facility, Rutherford Appleton Laboratory, STFC, Chilton, Didcot, OXON OX11 0QX, UK*

[3] *Max-Planck-Institut für Festkörperforschung, Heisenbergstraße 1, D-70569 Stuttgart, Germany*

[4] *Max Planck Society Outstation at the Heinz Maier-Leibnitz Zentrum (MLZ), D-85748 Garching, Germany*

* Corresponding author: thomas.ederth@liu.se

Link to main text:
https://doi.org/10.1021/acs.langmuir.1c02784

X-ray reflectivity data

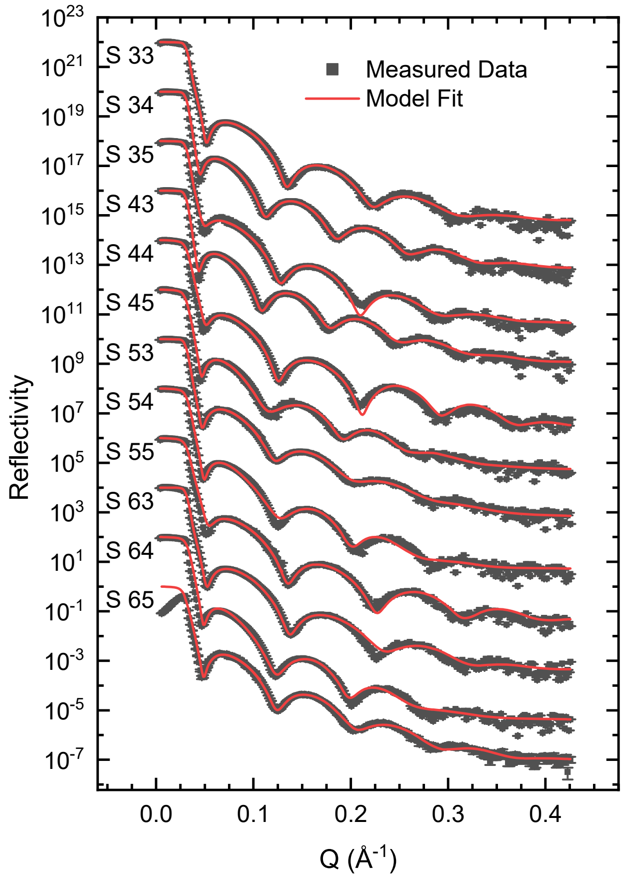

Figure S1. Measured XRR curves (symbols) with the corresponding **bi-sigmoidal model fits** (lines) before the deposition of the pAEMA layer. The errors are smaller than the symbols. Data set S65 is correctly positioned relative to the vertical axis. Subsequent data sets have been scaled x100 relative to the previous data set, for clarity.

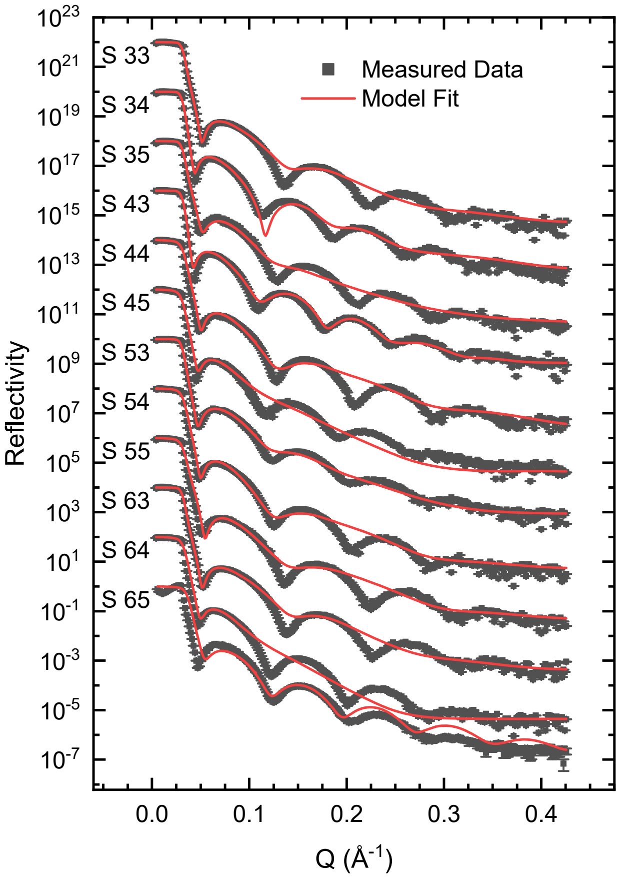

Figure S2. Measured XRR curves (symbols) with the corresponding **single block model fits** (lines) before the deposition of the pAEMA layer. The errors are smaller than the symbols. Data set S65 is correctly positioned relative to the vertical axis. Subsequent data sets have been scaled x100 relative to the previous data set, for clarity.

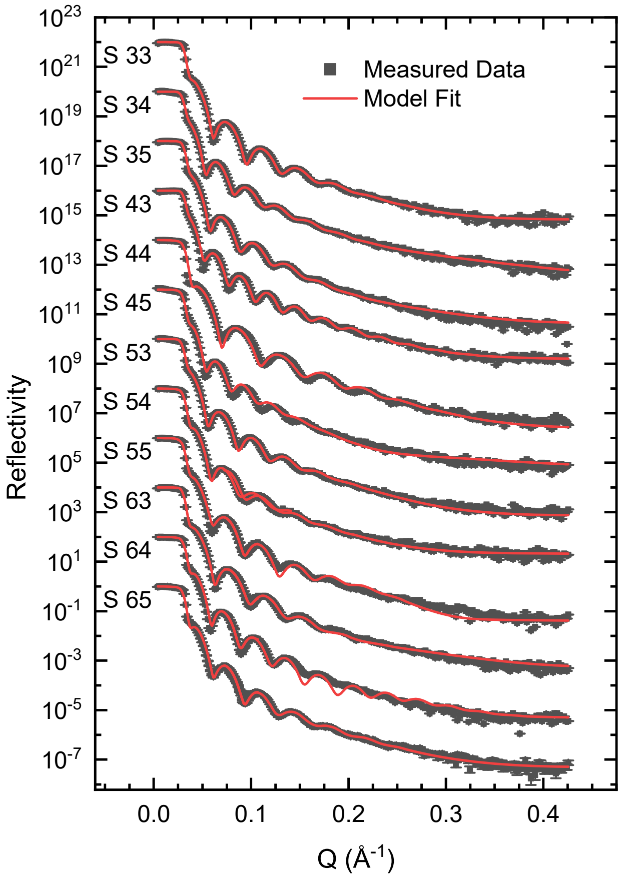

Figure S3. Measured XRR curves (symbols) with the corresponding **bi-sigmoidal model fits** (lines) after the deposition of the pAEMA layer. The errors are smaller than the symbols. Data set S65 is correctly positioned relative to the vertical axis. Subsequent data sets have been scaled x100 relative to the previous data set, for clarity

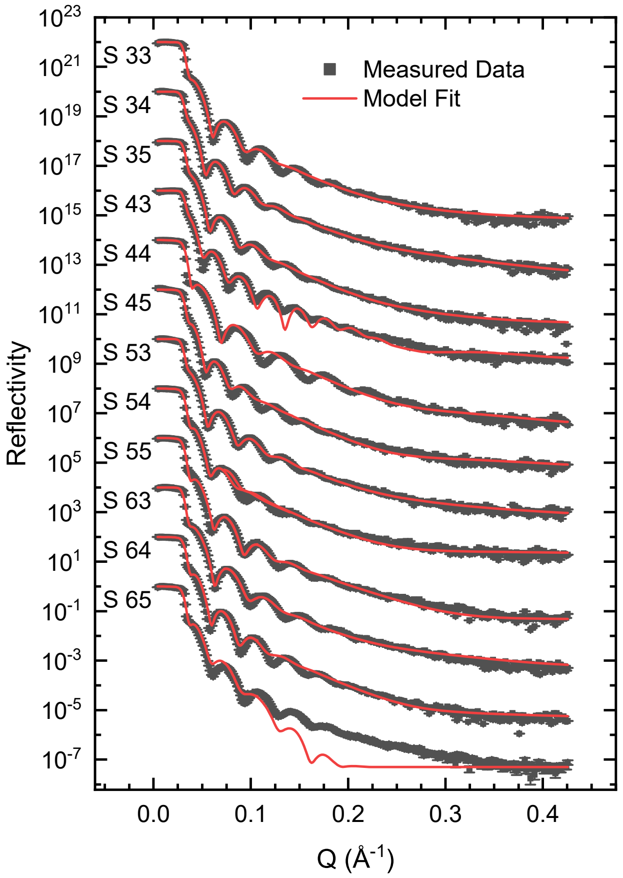

Figure S4. Measured XRR curves (symbols) with the corresponding **single block model fits** (lines) after the deposition of the pAEMA layer. The errors are smaller than the symbols. Data set S65 is correctly positioned relative to the vertical axis. Subsequent data sets have been scaled x100 relative to the previous data set, for clarity

Table S1. Parameters of the XRR model fits for the MAA layers, showing layer thicknesses (*d*), number densities (ρ) and interfacial roughnesses (σ). The sample names reflect the polymerization times of the two layers; sample "S*ab*" indicate *a* min pdMAA polymerization, and *b* min pAEMA polymerization. The subscript *dMAA* corresponds to the initial polymer layer, and the subscript *dMAA 2* refers to the layer with zero thickness which is used to adjust the interfacial roughness profiles. The polymer SLDs were calculated by multiplying the scattering length of a dMAA molecule with the densities presented in the table.

Sample	d_{SiO2} (Å)	σ_{SiO2} (Å)		d_{dMAA} (Å)	σ_{dMAA} (Å)	ρ_{dMAA} (10^{-3} Å^{-3})		$\sigma_{dMAA\ 2}$ (Å)	$\rho_{dMAA\ 2}$ (10^{-3} Å^{-3})
**S33**	11 ± 3	3.5 ± 1.5		69.8 ± 0.7	6.8 ± 1.1	10.4 ± 0.3		35 ± 4	6.0 ± 0.4
**S34**	10 ± 2	3.6 ± 0.7		84.2 ± 0.8	6.5 ± 1.2	9.4 ± 0.4		23 ± 2	5.3 ± 0.5
**S35**	10.0 ± 0.9	7.0 ± 0.2		74.1 ± 0.4	3.2 ± 0.5	9.90 ± 0.18		35 ± 5	7.37 ± 0.05
**S43**	10 ± 4	6.6 ± 1.1		87.3 ± 0.9	25 ± 4	9.7 ± 0.3		4.4 ± 0.6	7.6 ± 0.4
S44	*21 ± 5*	*4.7 ± 1.4*		*76.3 ± 1.5*	*5 ± 2*	*9.5 ± 0.6*		*31 ± 6*	*5.9 ± 0.5*
**S45**	32 ± 10	8 ± 3		84.0 ± 2.0	5.1 ± 1.2	10.0 ± 0.6		48 ± 19	4.2 ± 0.6
**S53**	11 ± 3	8.0 ± 1.0		76.5 ± 0.7	35 ± 8	9.8 ± 0.3		5.1 ± 0.4	7.8 ± 0.2
**S54**	35 ± 8	8 ± 3		80 ± 2	5.8 ± 1.4	10.1 ± 0.8		50 ± 21	4.7 ± 0.7
**S55**	18.1 ± 0.9	5.8 ± 0.3		70.7 ± 0.4	30 ± 2	9.66 ± 0.05		3.0 ± 0.5	2.78 ± 0.12
**S63**	16 ± 3	6.7 ± 1.0		67.9 ± 0.7	4.7 ± 0.9	10.1 ± 0.3		42 ± 7	5.5 ± 0.3
**S64**	35 ± 7	9 ± 3		81 ± 3	5.5 ± 1.8	9.5 ± 0.9		25 ± 26	5.1 ± 0.8
**S65**	15 ± 2	6.3 ± 0.8		75.9 ± 0.9	5.1 ± 0.5	9.0 ± 0.5		4.9 ± 1.5	2 ±7

Table S2. Parameters of the XRR model fits for the co-polymer layers, showing layer thicknesses (*d*), number densities (ρ) and interfacial roughnesses (σ). The sample names reflect the polymerization times of the two layers; sample "S*ab*" indicate *a* min pdMAA polymerization, and *b* min pAEMA polymerization. The subscript *cP* refers to the co-polymer layer after subsequent grafting of p(AEMA), considered together, and the subscript *cP 2* refers to the layer with zero thickness which is used to adjust the interfacial roughness profiles. The polymer SLDs were calculated by multiplying the scattering length of a dMAA molecule with the densities presented in the table.

Sample	d_{SiO2} (Å)	σ_{SiO2} (Å)		$d_{cP\ 1}$ (Å)	$\sigma_{cP\ 1}$ (Å)	$\rho_{cP\ 1}$ (10^{-3} Å^{-3})		$\sigma_{cP\ 2}$ (Å)	$\rho_{cP\ 2}$ (10^{-3} Å^{-3})
**S33**	16 ± 6	12.6 ± 1.6		168.9 ± 1.9	5.0 ± 0.8	10.2 ± 0.3		29 ± 3	6.2 ± 0.3
**S34**	15 ± 3	3.6 ± 0.8		195.9 ± 2.0	25.0 ± 1.7	10.0 ± 0.2		13 ± 3	2.8 ± 0.9
**S35**	16 ± 3	4.9 ± 0.7		183.0 ± 1.6	15.7 ± 1.6	10.4 ± 0.2		34 ± 4	5.2 ± 0.6
**S43**	16 ± 7	9 ± 3		217 ± 3	24 ± 3	11.1 ± 0.5		4.8 ± 0.7	5.9 ± 0.5
S44	*30 ± 6*	*11 ± 3*		*149 ± 4*	*4.7 ± 1.4*	*10.0 ± 0.6*		*41 ± 10*	*5.9 ± 0.6*
**S45**	16 ± 6	3.7 ± 1.8		214 ± 7	18 ± 5	11.1 ±0.3		50 ± 12	6.0 ± 1.5
**S53**	11 ± 9	14.6 ± 1.8		184.7 ± 1.9	4.9 ± 1.0	9.6 ± 0.3		27 ± 3	4.9 ± 0.3
**S54**	35 ± 6	16 ± 3		186 ± 4	4.9 ± 1.5	10.8 ±0.4		45 ± 10	6.4 ± 0.4
**S55**	30.2 ± 1.2	5.8 ± 0.4		176.6 ± 0.8	44 ± 20	10.00 ± 0.10		11.9 ± 0.6	4.04 ± 0.19
**S63**	17 ± 3	4.2 ± 1.1		162.2 ± 1.9	14.0 ± 1.8	10.3 ± 0.3		32 ± 4	5.6 ± 0.7
**S64**	22 ± 5	5.0 ± 1.8		188 ± 3	8 ± 6	10.6 ± 0.5		25 ± 7	8.7 ± 0.6
**S65**	24.3 ± 2.0	12.1 ± 0.6		176.3 ± 0.8	4.6 ± 0.3	11.13 ± 0.11		27.7 ± 1.0	7.45 ± 0.09

Bi-sigmoidal roughness

Mathematical models of random surfaces are commonly assigned a Gaussian distribution. There is good reason to assume that this is based more on mathematical convenience than empirical evidence, since it has been recognized for long [see e.g., P. Beckmann, “Scattering by non-Gaussian surfaces” *IEEE Transactions on Antennas and Propagation,* 21(2) 169 - 175, (1973), DOI: 10.1109/TAP.1973.1140444] that many practical surfaces are non-Gaussian. Thus, non-Gaussian models for surfaces or interfaces should perhaps be more common than they are in the literature. The bi-sigmoidal model is merely one such model, which allows the interfacial profile to be modulated, by introducing an additional parameter which permits adjustment of the sharpness of the interface (kurtosis) with little change in the thickness of the transition region. With a single-parameter sigmoid function, the sharpness of the profile and the thickness of the transition region are directly dependent on each other, while these are de-coupled in the bi-sigmoidal model.

To implement this model in the GenX software, an additional layer was defined on top of the original polymer layer, with its thickness constrained to zero and its volume density below the nominal volume density of the MAA monomer. To illustrate the resulting changes to the sigmoidal we have simulated two series of SLD profiles on a d = 100 Å thick layer with SLD values $\rho_1 = 1.0 \times 10^{-6}$ Å^{-2} and $\rho_2 = 0.2 \times 10^{-6}$ Å^{-2} and roughness values σ_1 = 10 Å and σ_2 = 10 Å. In these series we have systematically changed ρ_2 and σ_2 to demonstrate how the parameters influence the shape of the interface. The resulting interfaces are displayed in Figure S5, along with the original sigmoidal profiles, for comparison. Integrating the profiles show that the total amount of polymer is the same in all cases where $\rho_1 > \rho_2$. This was achieved by limiting the two number densities during fitting to be below (ρ_2) and above (ρ_1) the nominal number density of pMAA.

Whether an interfacial roughness function is able to ‘smear out’ material if it is adjacent to a zero-thickness layer on one side depends on the mathematical implementation. If only the material available within the slabs adjacent to the interface can be smeared out, no material will be smeared if the layer thickness is zero, but if the implementation smears out whatever material is available within the extent of the roughness profile, this is permissible.

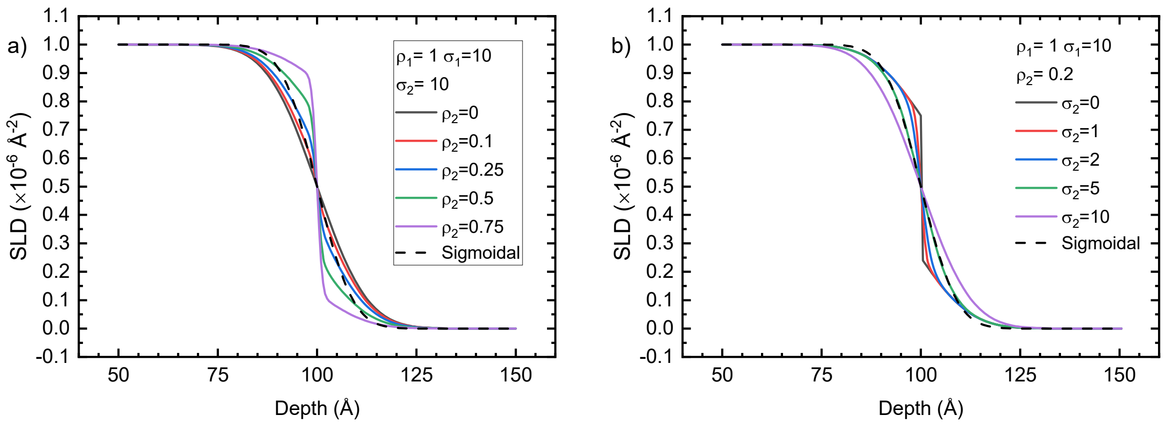

Figure S5: Simulated bi-sigmoidal SLD profiles with different ρ_2 values (a) and different σ_2 values (b). For comparison the sigmoidal profile is also displayed (dashed line).

Comparison of the one-layer and the two-layer models for NR

Table S3. The thicknesses and the dMAA contents for the entire polymer films, as obtained from models treating the polymer as a one-layer slab or a two-layer slab. The values are obtained from averaged data, where all samples with the same AEMA grafting time were considered together.

AEMA grafting time (min)	Total thickness (Å)		dMAA content (%)	
	1-layer model	2-layer model	1-layer model	2-layer model
3	172 ± 9	156 ± 10	36.0 ± 1.4	38.5 ± 1.9
4	149 ± 11	123 ± 9	34.2 ± 0.9	37.0 ± 1.6
5	158 ± 8	168 ± 8	32.9 ± 0.7	22.8 ± 1.1

Table S4. The Figure of Merit (FoM) values for the one-layer and the two-layer slab models, respectively.

Sample	S33	S34	S35	S43	S44	S45	S53	S54	S55	S63	S64	S65
1-layer	1.28	1.26	1.12	1.55	2.57	3.38	1.42	5.86	5.51	2.90	6.21	3.27
2-layer	0.87	1.24	0.97	1.17	1.04	2.40	1.19	1.28	2.86	1.22	1.57	1.56

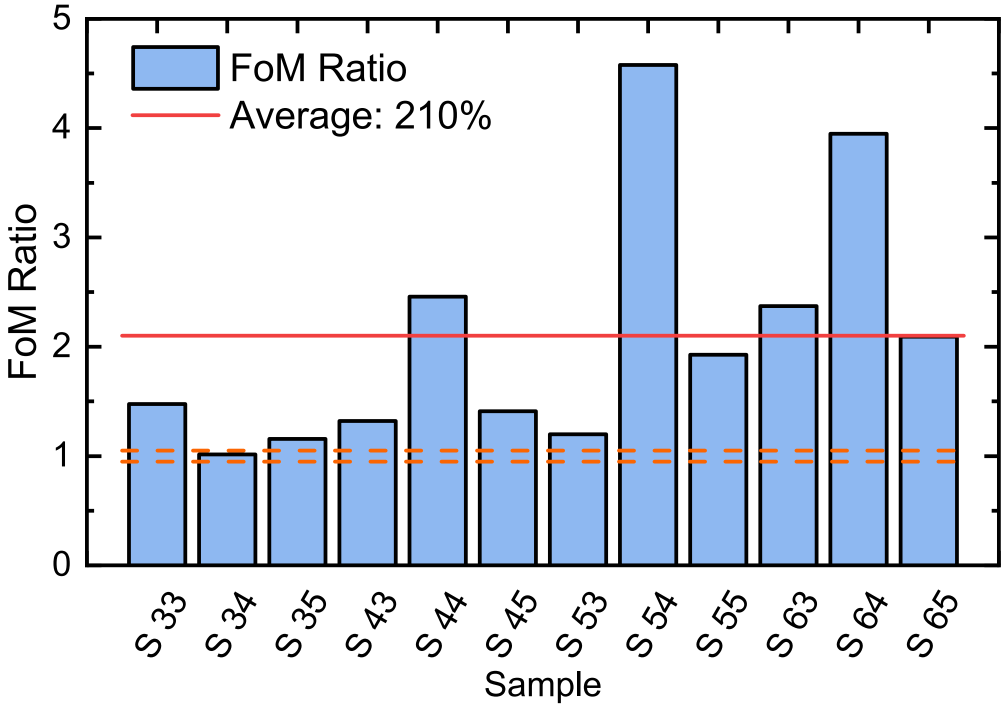

Figure S6. The ratios of the Figures of Merit (bars) from fitting to the one-layer and two-layer models, from Table S2 are plotted for each sample, for comparison. The average increase in FoM was found to be 110% (red line), on switching from a 2-layer to a 1-layer model. The dashed orange lines represent the ±5% FoM increase interval corresponding to the error estimation.

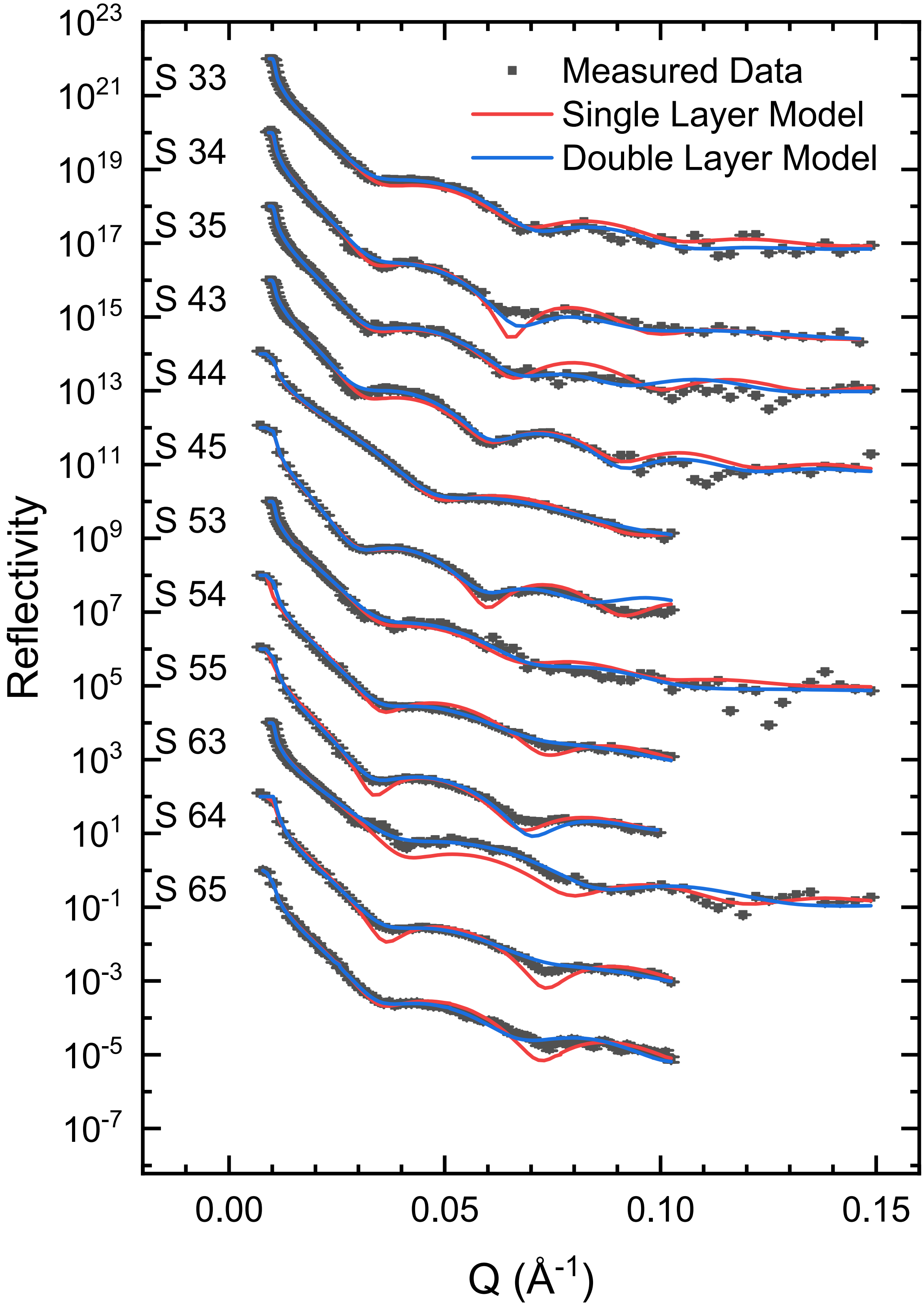

Figure S7. For comparison, the fits to the single- (red) and double-layer (blue) models are presented for the samples. Data set S65 is correctly positioned relative to the vertical axis. Subsequent data sets have been scaled x100 relative to the previous data set, for clarity

The model used for fitting the spectroscopic ellipsometry data

The same types of models were used for fitting to both the thickness data (d), and to the water volume fraction (VF) data. The expressions for the "U curve" models are

$$\left.\begin{aligned} d &= d_{max}\left(1 - \Delta d\left(erf\left(\frac{x - x_{pH-}}{w_{pH-}}\right) - erf\left(\frac{x - x_{pH+}}{w_{pH+}}\right)\right)\right) \\ VF &= VF_{max}\left(1 - \Delta VF\left(erf\left(\frac{x - x_{pH-}}{w_{pH-}}\right) - erf\left(\frac{x - x_{pH+}}{w_{pH+}}\right)\right)\right) \end{aligned}\right\}, \tag{S1}$$

where $x_{pH\mp}$ are the values of the lower or higher transition pH, respectively, $w_{pH\mp}$ is the slope of the transitions, d_{max} and VF_{max} are the maximum values for either the swollen thickness or the water volume fraction, and Δd and ΔVF are the changes in the swollen thickness or the water volume fraction for a given sample. During the modelling, the values of the transition pH and the transition slopes were constrained to be the same for both the swollen thickness and the volume fraction models for each sample. Furthermore, the d_{max} and VF_{max} values were constrained to the maximum of the measured data. Similarly, the "S curve" models are

$$\left.\begin{aligned} d &= d_{max}\left(1 - \Delta d\left(1 - erf\left(\frac{x - x_{pH+}}{w_{pH+}}\right)\right)\right) \\ VF &= VF_{max}\left(1 - \Delta VF\left(1 - erf\left(\frac{x - x_{pH+}}{w_{pH+}}\right)\right)\right) \end{aligned}\right\}. \tag{S2}$$

Figure S8. (Over the following three pages) Thickness and water volume fractions measured with spectroscopic ellipsometry at different pH values, for all samples except S54 and S55. One of two types of sigmoidal models were fitted to the data, depending on the number of transitions in the curve.

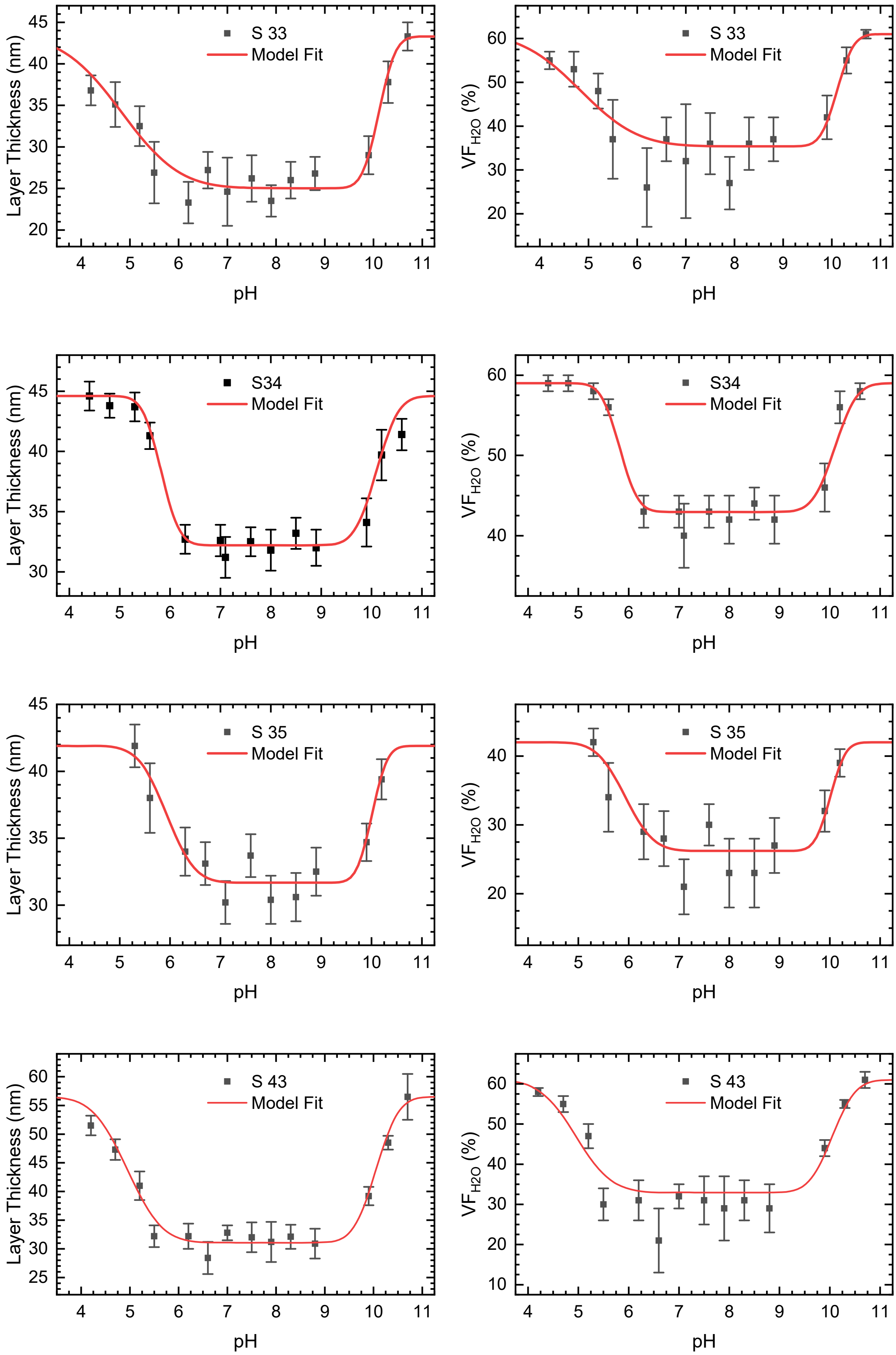
Layer Thickness (nm)
S 33
Model Fit
pH
VF_{H2O} (%)
S 33
Model Fit
pH
Layer Thickness (nm)
S34
Model Fit
pH
VF_{H2O} (%)
S34
Model Fit
pH
Layer Thickness (nm)
S 35
Model Fit
pH
VF_{H2O} (%)
S 35
Model Fit
pH
Layer Thickness (nm)
S 43
Model Fit
pH
VF_{H2O} (%)
S 43
Model Fit
pH

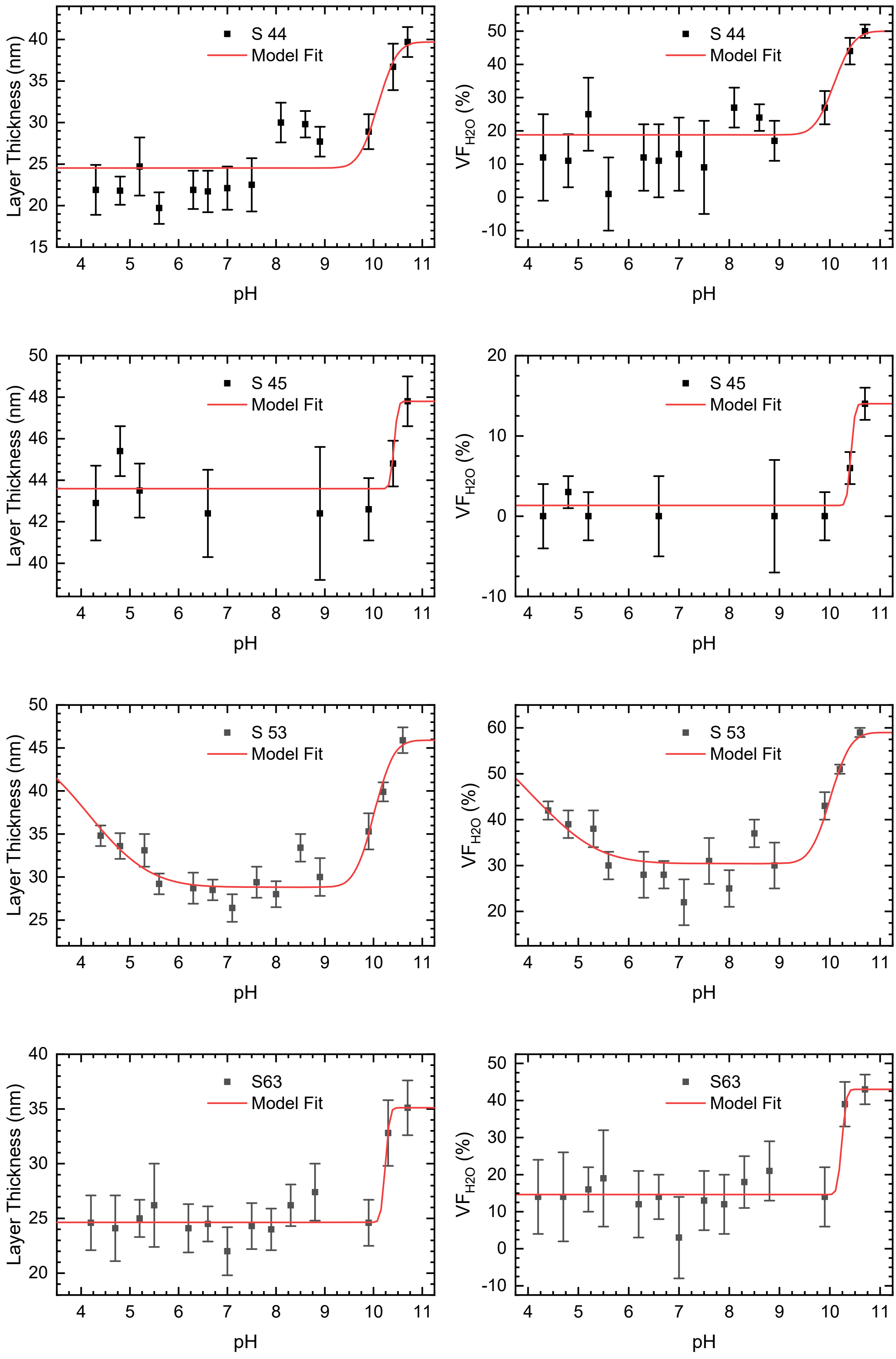
S 44
Model Fit
Layer Thickness (nm)
VF_{H2O} (%)
pH
S 45
Model Fit
Layer Thickness (nm)
VF_{H2O} (%)
pH
S 53
Model Fit
Layer Thickness (nm)
VF_{H2O} (%)
pH
S63
Model Fit
Layer Thickness (nm)
VF_{H2O} (%)
pH

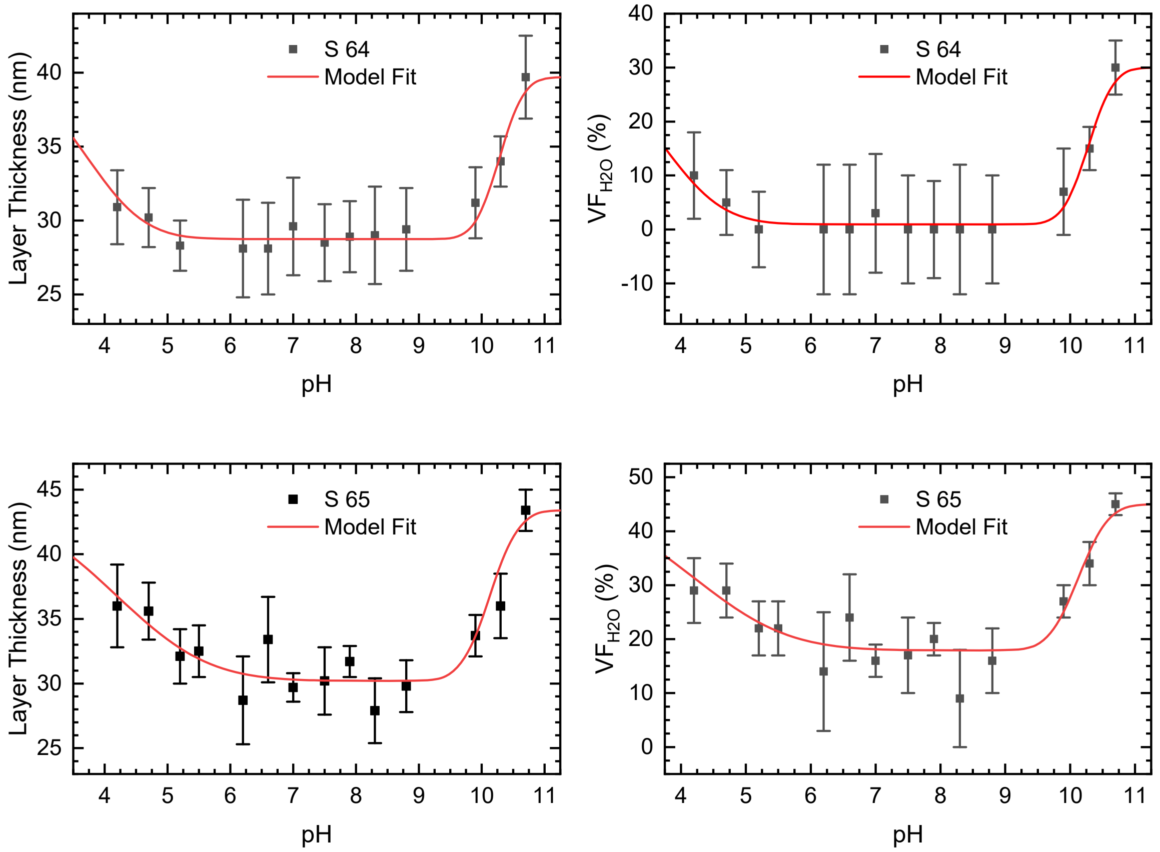
Layer Thickness (nm)
pH
S 64
Model Fit
VF$_{H2O}$ (%)
pH
S 64
Model Fit
Layer Thickness (nm)
pH
S 65
Model Fit
VF$_{H2O}$ (%)
pH
S 65
Model Fit
